# Empirical optimization of an angled spoke paddling wheel with self-rotating mechanism

**DOI:** 10.1038/s41598-022-25181-7

**Published:** 2022-11-28

**Authors:** Chaewon Kim, Seungkyu Han, Jeeho Won, TaeWon Seo

**Affiliations:** School of Mechanical Engineering, Hanyang University, Seoul, 04763 South Korea

**Keywords:** Engineering, Mechanical engineering

## Abstract

The development of the maritime industry has led to a corresponding increase in maritime accidents. Maritime accidents are major events that are costly to recover and can cause casualties. Moreover, individuals who are brought to the scene for recovery or rescue are at risk. To tackle this issue, the wheel mechanism of a water rescue robot, i.e., the angled spoke paddling wheel (ASPW), has been studied. The purpose of this study is to optimize the paddle design parameters of the ASPW using the Taguchi method. Experiments are conducted by creating paddles with various combinations of design parameters using ${\textbf{L}}_9$($3^4$) orthogonal arrays. The objective function is determining the optimal combination of paddle design parameters that will produce the greatest thrust force at the same RPM. Sensitivity analysis of each design parameter is conducted by calculating the signal-to-noise ratio from the experimental results. The pitch angle is found to be the most sensitive parameter. An additional experiment is conducted based on the results of the sensitivity analysis. The results show that the optimal design parameters are a pitch angle of $0^{\circ }$, rectangular end shape, X-axis curvature of 37.5 mm, and Y-axis curvature of 25 mm. The paddle with this combination of design parameters have a maximum thrust force of 64.74 gf at 120 RPM and exhibit up to an 18.27% improvement in performance compared with the initial paddle before optimization.

## Introduction

Most of the Earth’s surface is covered with water. Consequently, resources that are difficult to obtain on land are more easily accessible from the sea and present in larger quantities. In addition, the sea serves as a link between continents and countries. Therefore, the maritime industry has a commercial advantage that is closely related to economic development^1^. Many countries are developing their maritime industry as evidenced by the steady increase in sea trade^2^.

However, the increase in maritime trade has been accompanied by an increase in the number of maritime accidents. Statistics show that cargo ships, i.e., general cargo and bulk carriers, account for the highest proportions of maritime accidents at 21% and 31%, respectively. Between 2003 and 2007, amidst the booming shipping industry, maritime accidents surged and the number of bulk-carrier fleets at risk increased by 230%. In addition, 57% of accidents occurred in ports close to land^3^. Maritime accidents are very serious because they not only cause economic losses but can also lead to casualties. The presence of mobile robots at the scene of an accident can assist in rescue and recovery operations to significantly reduce the risks faced by rescuers.

Several studies have been conducted on amphibious robots^4^. The AmphiHex-I^5^, AmphiHex-II^6^, SeaDog^7^, and Claw-Wheel^8^ achieved amphibious driving by transforming the shape of their wheels into something other than a closed circle. The amphibious system of an eccentric paddle-based robot^9–12^ was realized by rotating the paddle using eccentricity. The wheel-propeller-leg^13^ and AmphiSTAR^14^ robots can be driven on both land and water because they use a propeller rather than a wheel. Robots that are not driven by rotational propulsion include the six-legged ALUV^15^, snake-like robot ACM-R5^16,17^, centipede-like robot Ambot^18^, and turtle-like robot ASRobot^19^. However, these robots are not suitable for rescue operations owing to their disadvantages. The wheel-propeller-leg^13^ and AmphiSTAR^14^ robots exhibit high-speed performance on both the water surface and ground, but their ability to avoid obstacles is limited. The ALUV^15^, ACM-R5^16,17^, and ASRobot^19^ are too slow to function as rescue robots. The AmphiHex-I^5^, AmphiHex-II^6^, SeaDog^7^, Claw-Wheel^8^, Ambot^18^, and eccentric paddle-based robot^9–12^ rotate like a wheel, which causes the wheel-rotation trajectory to rise above the water surface. This obstructs the view of the robot camera and makes it difficult to enter low-height spaces.

**Figure 1:**
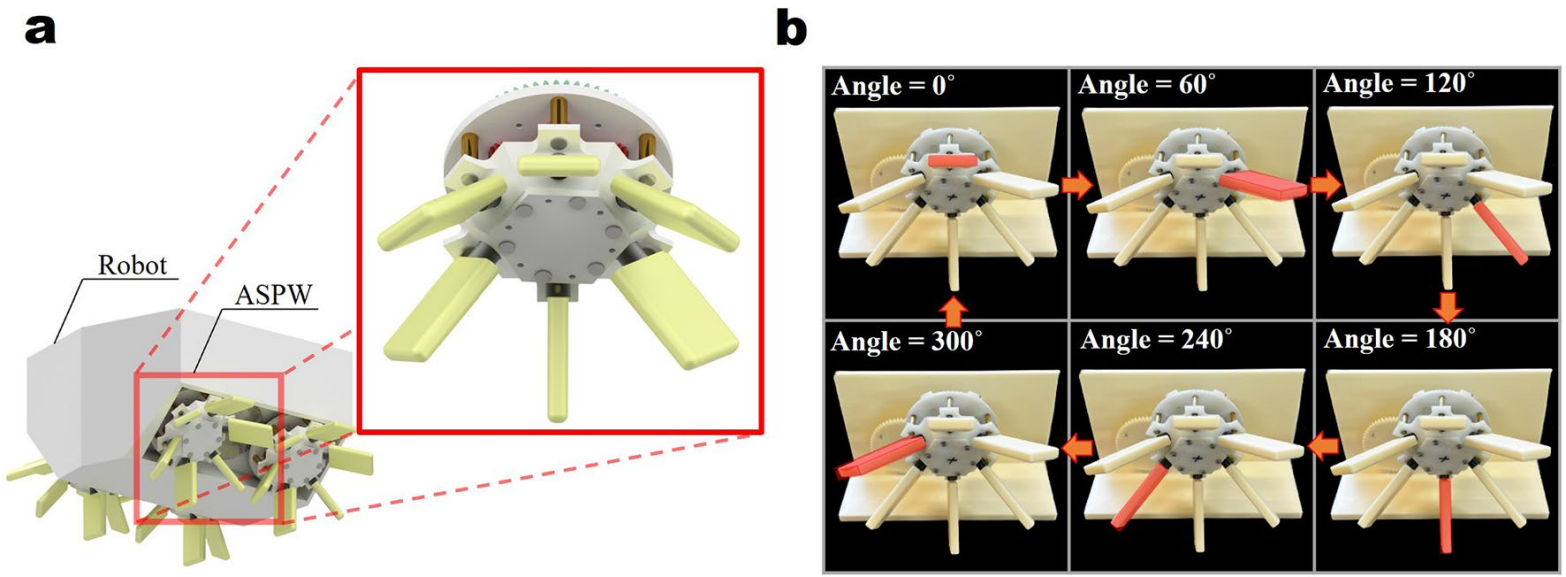
(**a**) Robot in which the ASPW is installed. (**b**) Rotation sequence of one paddle of the ASPW.

In this study, the paddle design of the angled spoke paddling wheel (ASPW) is optimized. The ASPW is a new amphibious mechanism that can deal with the disadvantages by using planetary and external gears. Additionally, it has a high driving speed and the ability to overcome obstacles^20^. The ASPW is attached to the amphibious robot, as shown in Fig. 1a. A paddle of the ASPW moves, as shown in Fig. 1b, when rotating. The paddle rotates 30$^{\circ }$ as it revolves 60$^{\circ }$ owing to the gears inside the ASPW. The top and bottom paddles form a right angle and move with a rowing motion. The detailed configuration and mechanism of the ASPW are described in “The description of ASPW” section. Because the trajectory of the ASPW is only generated at the lower part of the robot body, it is convenient to use the ASPW when the sensor or camera is attached to the robot body. Furthermore, it can pass through low-height spaces.

Previous studies have determined the most effective propeller design by simulation or experiments. A remotely operated vehicle was analyzed by simulation after 3D modeling by controlling the parameters affecting the thrust force, and the results were validated experimentally^21^. The performance of a marine propeller was evaluated by a computer program and the parameters of different models were compared^22^. The flow velocity around the propeller was predicted by considering the viscosity of the fluid, whereas the thrust force of the propeller was numerically predicted^23^. The performance of a typical propeller, design optimization, and thrust force prediction of paddles or blades were verified experimentally after optimization through simulation or numerical analysis.

Unlike a typical propeller, the ASPW does not continuously rotate; neither does it move with a typical paddling motion. Instead, it combines these two motions. Some sections of the ASPW rotate like a propeller, whereas other sections move with a typical paddling motion. These movements are neither constant nor static. Because of this characteristic, the area and shape at which the paddle collides with the water is not constant during one rotation cycle. Therefore, it is difficult to analyze and optimize the performance of the ASPW using simulation. In this study, we attempt to determine the optimal design parameters of the ASPW paddle experimentally.

The Taguchi method was used in the experimental study of paddle design optimization. The Taguchi method is a design technique that optimizes a design by considering the external factors that are difficult to control. Because the optimal design parameters are selected through experiments at the design stage, the desired product or robot can be implemented quickly and inexpensively. Moreover, even if the same number of experiments are performed, enhanced design variable values can be obtained and the quality of the results is improved^24,25^. An example of effective optimization using the Taguchi method is sand-running mobile robot. Since sand has both solid and fuild characteristics, it is difficult to optimize through theoretical methods. This sand-running mobile robot enables efficient running by reducing slip in sand by optimizing the shape of the robot leg through the Taguchi method^26^. Like this case, ASPW will also be able to optimize the shape by using the Taguchi method. The purpose of this study is to maximize the driving performance of the ASPW using the Taguchi method and optimizing the paddle shape.

The remainder of this study is organized as follows. “The description of ASPW” section briefly describes the configuration and mechanism of the ASPW. “Experimental setup” section describes the test bench setup, and defines the design variables and user conditions for applying the Taguchi method. “Results and discussion” section describes the experiments performed, and presents the results and analysis. Finally, “Conclusion” section presents the conclusions of the study.

## The description of ASPW

The ASPW is an amphibious wheel that uses the wheel trajectory of the angled spoke wheel (ASW). This section describes the configuration and mechanism of the ASPW.

### Configuration of ASPW

**Figure 2:**
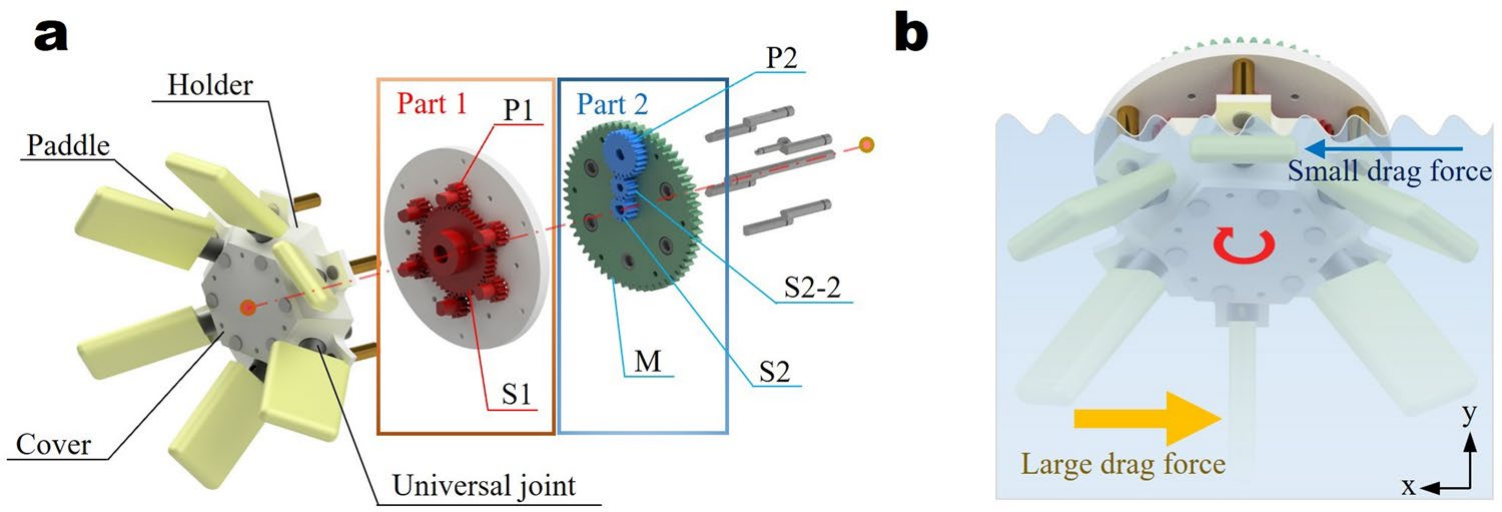
(**a**) Shows configuration of the ASPW. (**b**) Describes how ASPW generates force that propels the robot forward. ASPW is assumed to be rotating clockwise and the robot moves in the opposite direction of the x-axis. The yellow and blue arrows indicate the amount of drag force created on the paddle.

Figure 2a shows the details of the parts and arrangement of the ASPW. All the parts, except the stainless-steel shaft, brass support, and universal joint, were printed by a Stratasys 170 3D printer with acrylonitrile styrene acrylate (ASA) filament. The ASPW consists of a total of six gears: sun gears, planetary gears, and external gears. Part 1 consists of one sun gear S1 and six planetary gears, P1, that enable the rotation of the paddles. Part 2 consists of one sun gear S2 and two external gears, S2–2 and P2, that determine the orbital cycle of the paddles. The motor supplies power to gear M to operate the ASPW. A universal joint is connected to P1 and the paddle is coupled to this universal joint. As P1 rotates, both the universal joint and paddle rotate. The holder fixes the universal joint such that it can rotate at a constant angle as the ASPW operates. The cover fixes the holders.

### Mechanism of ASPW

The shaft in the middle is fixed. Gear M receives power from the motor, which is transmitted to the gears of the ASPW. Gears S2, S2–2, and P2 have a ratio of 1:1:2. This makes the ASPW’s top and bottom paddles at a right angle with each other. Gear S1 has 48 teeth, whereas P1 has 12 teeth. This causes the gears of Part 1 to rotate at an angle of 30$^{\circ }$ to each other when the ASPW revolves. Consequently, all paddles connected to the universal joint rotate at a constant 30$^{\circ }$ angle to each other as the paddles revolve by 60$^{\circ }$. By maintaining this phase, a large drag is generated when the paddle is at the bottom of the wheel, whereas a small drag is generated when the paddle is at the top of the wheel. This movement creates a propulsive force that is similar to rowing motion, as shown in Fig. 2b.

## Experimental setup

This section describes the settings and parameter selection for the experiment.

**Figure 3:**
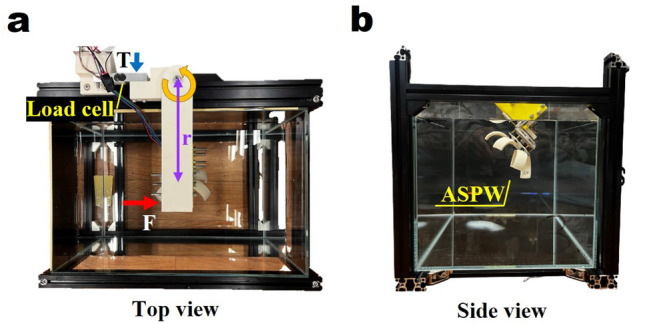
(**a**) Top view and the ASPW thrust force measurement method using a load cell. (**b**) Side view.

### Test bench configuration

Figure 3 shows the test bench configuration. Because the ASPW was tested while submerged in water, a clear glass water container measuring 0.4 m $\times$ 0.26 m $\times$ 0.3 m was used. An aluminum profile frame was built around the water container to attach the ASPW, sensors, boards, and motors. Other parts for fixing the motor, ASPW, and load cell were 3D printed using Stratasys 170, with ASA filament as the printing material. The motor used to drive the ASPW is the planetary geared motor IG-32GM 05TYPE with a 2-channel encoder; the motor driver is MD10C. Power was stably supplied through the power supply. A load cell was used to measure the thrust force of the ASPW. The HX711 amplifier was used for precise measurements.

As the motor drives and transmits power to the ASPW, the ASPW rotates and generates a thrust force with the paddle. This causes the beam connected to the ASPW to receive a force from the ASPW, which is converted into torque. The torque is measured by the load cell connected to the beam. In paddling, the interest is propulsive efficiency and drag force is the main factor. And the drag force generated by the paddle is utilized as a forward thrust force. Therefore, in this study, the thrust force was calculated using the torque measured in the load cell and used as the standard to evaluate the performance of paddles.

1 $$\begin{aligned} T = rF, \end{aligned}$$

where *T* is the torque, *r* is the length of the beam, and *F* is the thrust force.

### Using the Taguchi method

In this section, the design parameters and user conditions are set for the adoption of the Taguchi method. Subsequently, a suitable orthogonal array is selected and the combination of design parameters for testing is determined.

#### Setting design parameters and user conditions

**Figure 4:**
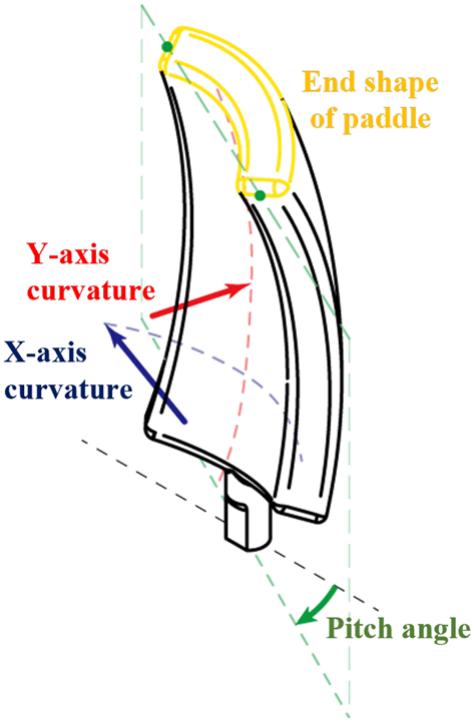
Design parameters of ASPW.

The design parameters and user conditions of the paddle must be selected to utilize the Taguchi method. There are four design parameters: pitch angle, end shape of the paddle, X-axis curvature, and Y-axis curvature. The pitch angle was selected based on research findings that the pitch angle of a kayak blade affects the performance. The X- and Y-axis curvatures were selected as parameters because of their influence on the change in the drag force coefficient was found in kayak blade performance experiments^27^. The end shape of the paddle was chosen because the drag force coefficient is dependent on the cross-sectional shape^28^. The four paddle design parameters are shown in Fig. 4. Each parameter was divided into three levels at regular intervals.

To determine the optimal combination of paddle design parameters that will generate the greatest thrust force at the same RPM, the user conditions were set to three levels: 60 RPM, 90 RPM, and 120 RPM. The paddles were evaluated based on the thrust force generated in each user condition. The design parameters and user conditions are listed in Table 1.

**Table 1:** Design parameters and user conditions.

		Level 1	Level 2	Level 3
**Design parameter**				
A	Pitch angle ($^{\circ }$)	0	22.5	45
B	End shape of the paddle	$\blacksquare$	$\blacktriangle$	$\bullet$
C	X-axis curvature (mm)	$\infty$	15	30
D	Y-axis curvature (mm)	$\infty$	25	50
**User condition**				
E	Motor rpm (rev/min)	60	90	120

#### Select orthogonal array

In the Taguchi method, the design parameters are combined using an orthogonal array. This method makes it possible to find the optimal combination of design parameters with less effort as there is no need to test all parameter combinations. The types of orthogonal arrays are ${\textbf{L}}_4$($2^3$), ${\textbf{L}}_8$($2^7$), ${\textbf{L}}_12$($2^11$), and ${\textbf{L}}_16$($4^1$)^24^. In this experiment, because there are three levels and four types of design parameters, the ${\textbf{L}}_9$($3^4$) orthogonal array was selected. Different parameter combinations were tested three times at each RPM to measure the thrust force, which is the objective function.

The experimental results were analyzed using the signal-to-noise(S/N) ratio to determine the optimal design parameters. The three methods to analyze the S/N ratio are the “nominal-the-better”, “higher-the-better”, and “lower-the-better” methods. In this experiment, the greater the thrust force and objective function, the better the result; hence, the “higher-the-better” method was used.

2 $$\begin{aligned} S/N\, ratio = -10\log\left| \frac{(\frac{1}{y_1})^2+(\frac{1}{y_2})^2+ \cdots +(\frac{1}{y_n})^2}{n}\right| [dB], \end{aligned}$$

where $y_1$ and $y_2$ $\ldots$ $y_n$ denotes the result of the experiment, i.e., the thrust force measured by the load cell; and *n* is the number of experiments conducted for each design parameter combination.

## Results and discussion

Table 2 presents the design parameter combinations and experimental results obtained using the orthogonal array ${\textbf{L}}_9$($3^4$) of the Taguchi method. The thrust force was calculated by (1). The torque-measurement method is shown in Fig. 3a. The first and second columns of the table list the number of experiments and combinations of design parameter levels, respectively. The third column lists the thrust force tested three times for each user condition level. Each experimental result is a value obtained by RMS for 10 s. Only the force generated in the direction of movement of the ASPW was considered as a positive result and measured. Finally, the fourth column of the table lists the S/N ratios calculated by (2).

### S/N ratio analysis

**Figure 5:**
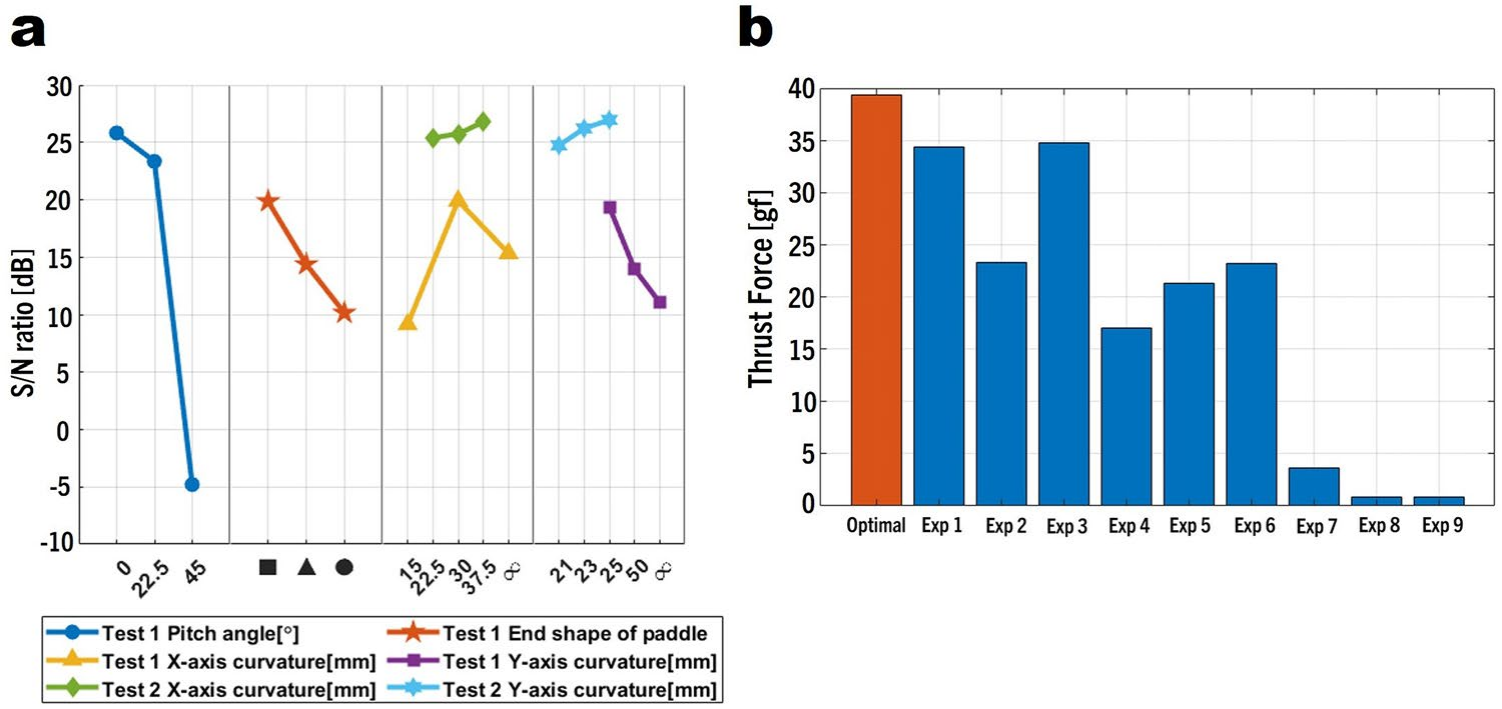
(**a**) Shows S/N ratio of ${\textbf{L}}_9$($3^4$) Taguchi orthogonal array. (**b**) Shows paddle performance comparison with optimal design parameters and other design parameters of the L9(34) orthogonal array. Exp 1 is the initial paddle design.

The S/N ratio indicates the sensitivity of a parameter. This means that the higher the S/N ratio of a parameter, the greater its influence on the experimental results. The lines corresponding to Test 1 in Fig. 5a are visual representations of the S/N ratios in Table 2. The most sensitive parameter was the pitch angle. At a pitch angle of 0$^{\circ }$, even when the paddle turned as the ASPW rotated, no force was generated in the direction opposite the direction of movement of the ASPW. As the pitch angle increased, the negative direction force increased corresponding to the positive direction force increase. Thus, it seems that the overall thrust force decreased. The sensitivities of the other three design parameters (i.e., end shape of the paddle, X-axis curvature, and Y-axis curvature) were similar. In the case of the end shape of the paddle, the best result was obtained when it was rectangular. This is because the drag coefficient was largest when the cross-sectional shape was rectangular. The X-axis and Y-axis curvatures generated the greatest thrust force at 30 and 25 mm, respectively. The drag coefficient varied depending on the curvature. It is believed that the largest drag coefficient occurred at these parameter values. Consequently, the combination of design parameters that generated the largest thrust force are a pitch angle of $0^{\circ }$, rectangular end shape of the paddle, X-axis curvature of 30 mm, and Y-axis curvature of 25 mm.

However, a paddle with these parameters is not yet the optimal design. This is because of the S/N ratio graph of the Y-axis curvature shows that 25 mm is not the highest point. There is a possibility that the S/N ratio will further increase when the radius of curvature is less than 25 mm. Similarly, for the X-axis curvature, a higher S/N ratio may exist at approximately 30 mm. To determine the optimal design parameters, additional experiments are required to adjust the design parameters.

### Optimal design parameters

Additional experiments were conducted to determine the optimal design parameters of the paddle. In the case of the pitch angle, the S/N ratio increased as it approached $0^{\circ }$. However, as the ASPW rotated, the paddle repeatedly switched between positive and negative angles. If the pitch angle is set to a negative angle, then the S/N ratio graph will appear symmetrical. Therefore, the optimal pitch angle is $0^{\circ }$. In the case of the end shape of the paddle, because the parameter values were not continuous, it was impossible to perform additional experiments. Therefore, the rectangular end shape was determined as the optimum shape. In the case of the X-axis curvature, the largest S/N ratio was obtained at 30 mm; however, an additional experiment was conducted to determine whether there is a parameter that will provide better results around this value. The parameter levels were set as 22.5, 30, and 37.5 mm as the median values of the previous level intervals. In the case of the Y-axis curvature, the S/N ratio increased as the radius approached 25 mm. Therefore, additional experiments should be conducted with a radius of curvature less than 25 mm. However, because of the paddle height of 42 mm, the radius of curvature cannot be less than 21 mm. Therefore, the Y-axis curvatures in the additional experiment were determined to be 21, 23, and 25 mm. Because there was no orthogonal array corresponding to the three levels of the two design parameters, all the cases were tested. The results of the additional experiment are summarized in Table 3.

**Table 2:** ${\textbf{L}}_9$($3^4$) Taguchi orthogonal array and experimental results.

Experiment number	Design parameter				User condition			S/N ratio (dB)
	A	B	C	D	E (gf)			
	Level				Level 1	Level 2	Level 3	
1	1	1	1	1	16.75	34.37	54.74	27.67
					15.64	33.99	53.84	
					15.55	33.61	54.55	
2	1	2	2	2	10.86	21.79	55.86	24.33
					11.08	23.27	53.74	
					10.15	22.88	53.33	
3	1	3	3	3	11.44	34.77	68.37	25.46
					12.49	31.49	66.39	
					11.1	32.49	64.88	
4	2	1	2	3	8.84	17.01	22.85	21.92
					8.69	14.87	21.67	
					8.61	15.99	20.67	
5	2	2	3	1	11.45	20.74	51.14	24.28
					10.28	21.3	48.68	
					10.86	21.08	49.73	
6	2	3	1	2	11.21	23.22	24.64	23.82
					10.8	22.5	20.74	
					10.49	21.79	23.35	
7	3	1	3	2	2.26	3.62	0	9.98
					2.28	2.95	0	
					2.36	2.63	0	
8	3	2	1	3	1.88	0.73	0.2	− 5.52
					1.3	0.85	0	
					1.62	0.77	0	
9	3	3	2	1	2.2	0.73	0.07	− 18.85
					1.36	0.89	0.05	
					1.64	0.75	0.09

**Figure 6:**
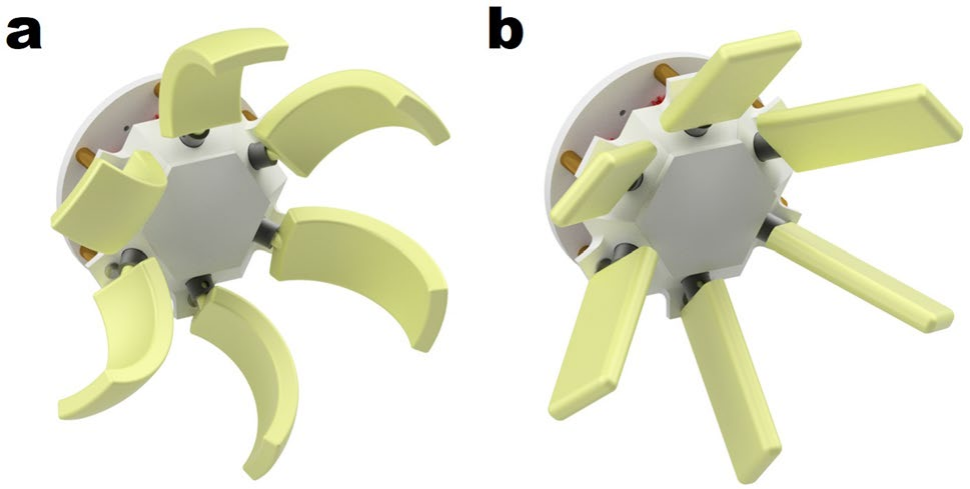
(**a**) ASPW using paddles with optimal design parameters. (**b**) ASPW using the initial paddle before optimization.

The lines corresponding to Test 2 in Fig. 5a show the S/N ratios in the additional experiment. The X-axis curvature that yielded the best result is 37.5 mm. Although it is possible to proceed with the experiment by setting a radius of curvature greater than 37.5 mm, because the curvature is already small and close to that of a flat surface, it was decided that additional experiments were unnecessary. In the case of the Y-axis curvature, the S/N ratio decreased as the radius of curvature decreased to 21 mm. Therefore, the optimal value was confirmed to be 25 mm. This optimal design parameters are listed in Table 3. The S/N ratio with this number of experiments is 27.91 dB, which is the highest among the experiments. Figure 5b compares the thrust force of the paddles at 90 RPM. The thrust force of the optimal paddle design was 64.74 gf at 120 RPM. In the case of the initial paddle, Exp 1, a thrust force of 54.74 gf was generated at the same RPM. Therefore, the performance of the optimized paddle improved by 18.27% compared with that of the initial paddle. In other user conditions, the thrust force improved by 14.58% at 90 RPM and remained almost unchanged at 60 RPM. Figure 6 compares the shapes of the optimized and initial paddles.

**Table 3:** Optimal design parameters by additional experiment.

Experiment number	Design parameter				Design parameter			S/N ration (dB)
	A	B	C	D	E (gf)			
	Level				Level 1	Level 2	Level 3	
Optimal	1	1	3	1	15.12	39.28	64.74	27.91
					16.73	39.38	64.46	
					16.15	35.98	64.43

## Conclusion

The purpose of this study was to develop a robust design for the paddle of the ASPW, a mechanism for amphibious locomotion that uses the wheel trajectory of the ASW. The ASPW is a new mechanism that has never been used in conventional amphibious robots, such as the simple rotation of propellers or paddles, which makes it difficult to perform theoretical mechanical analysis in fluids. A robust design of the ASPW paddle was achieved using the orthogonal array of the Taguchi method. There are four design parameters: pitch angle, end shape of the paddle, X-axis curvature, and Y-axis curvature; and three user conditions: 60 RPM, 90 RPM, and 120 RPM. The orthogonal array ${\textbf{L}}_9$($3^4$) was used. The experimental results revealed that the most sensitive design parameter was the pitch angle. The closer the pitch angle was to $0^{\circ }$, the stronger the thrust force generated. The sensitivities of the other parameters were similar. The optimal design parameters obtained experimentally are: pitch angle of $0^{\circ }$, rectangular end shape of the paddle, X-axis curvature of 37.5 mm, and Y-axis curvature of 25 mm. The S/N ratio of these optimal design parameters was 27.91 dB. The measured thrust was 64.74 gf at 120 RPM. The performance improved by 18.27% compared with that of the initial paddle at 120 RPM. At 90 RPM, the thrust force increased by 14.58%. At 60 RPM, the thrust force was relatively unchanged. The results of this study show that the Taguchi method is effective for the robust design of the paddle of the ASPW.

## Supplementary Information

Supplementary Information.

## Data Availability

All data generated or analysed during this study are included in this published article and its Supplementary Information files.
