# Supplementary figures and images for: Empirical optimization of an angled spoke paddling wheel with self-rotating mechanism
Source: Sci Rep. 2022 Nov 28;12:20511. doi: 10.1038/s41598-022-25181-7 (PMC9705443; doi:10.1038/s41598-022-25181-7)

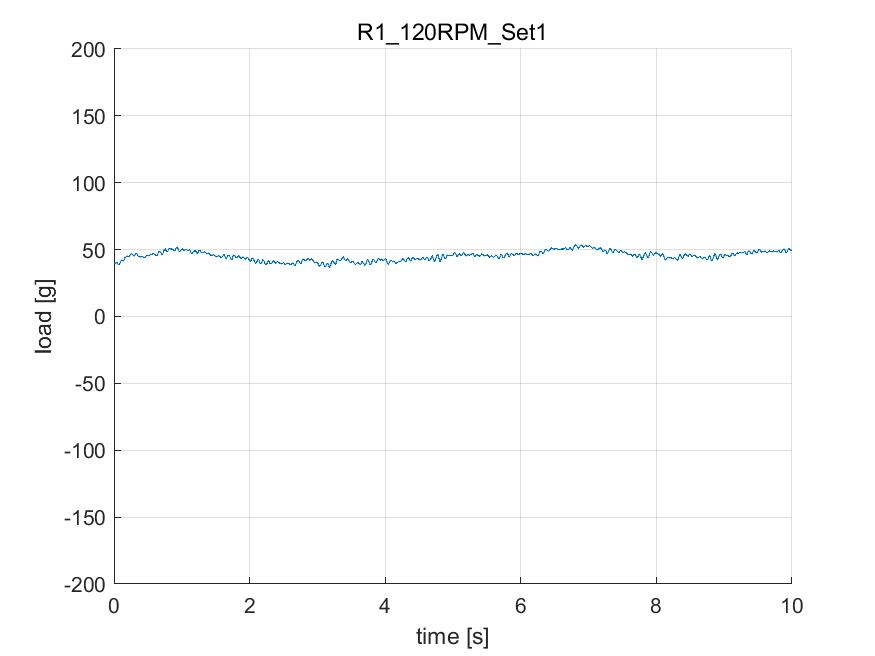

Supplement: Supplementary file 1 — Supplementary Information. [file 41598_2022_25181_MOESM1_ESM.zip › Loadcell_data_graph/R1_120RPM_Set1.png]

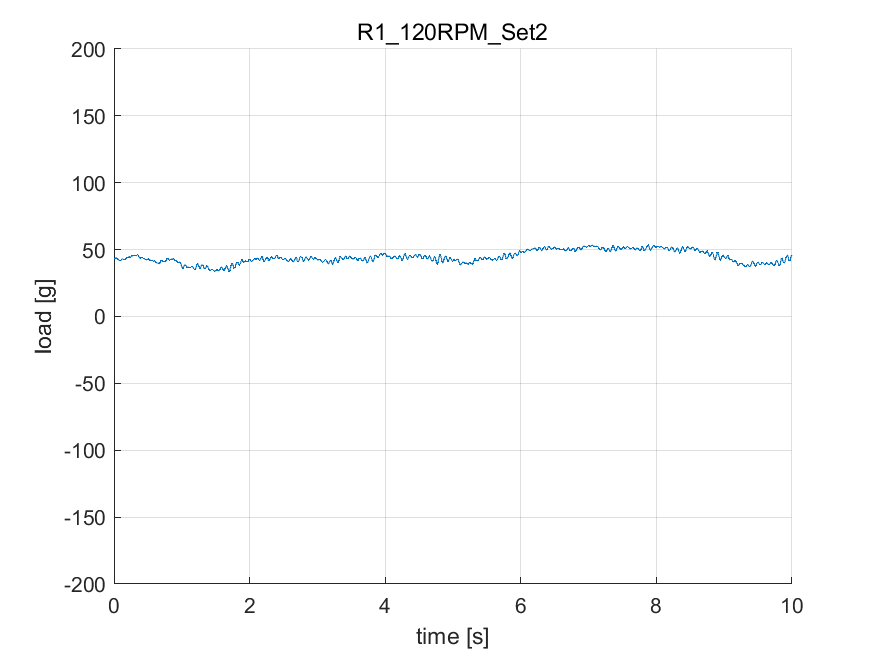

Supplement: Supplementary file 1 — Supplementary Information. [file 41598_2022_25181_MOESM1_ESM.zip › Loadcell_data_graph/R1_120RPM_Set2.png]

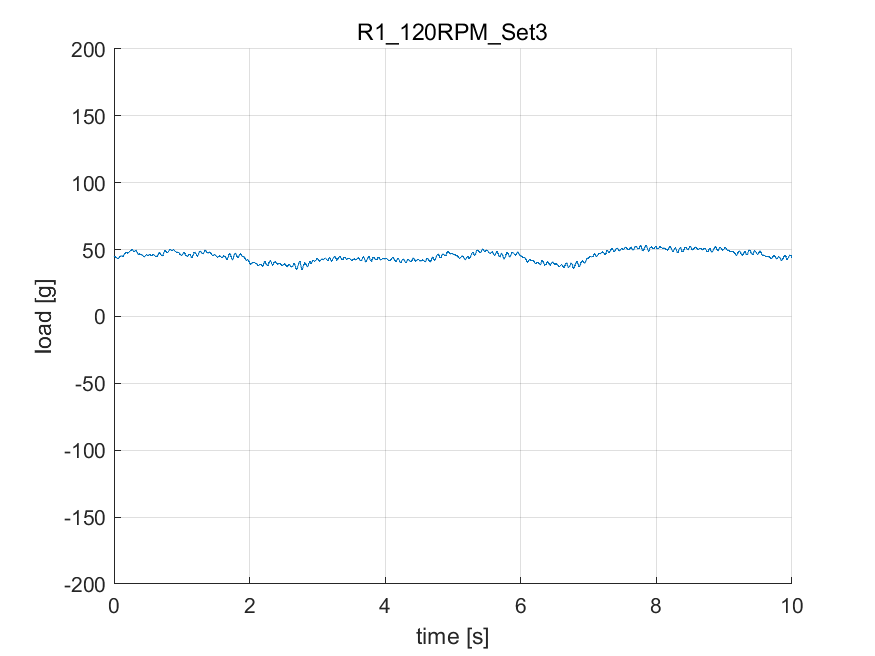

Supplement: Supplementary file 1 — Supplementary Information. [file 41598_2022_25181_MOESM1_ESM.zip › Loadcell_data_graph/R1_120RPM_Set3.png]

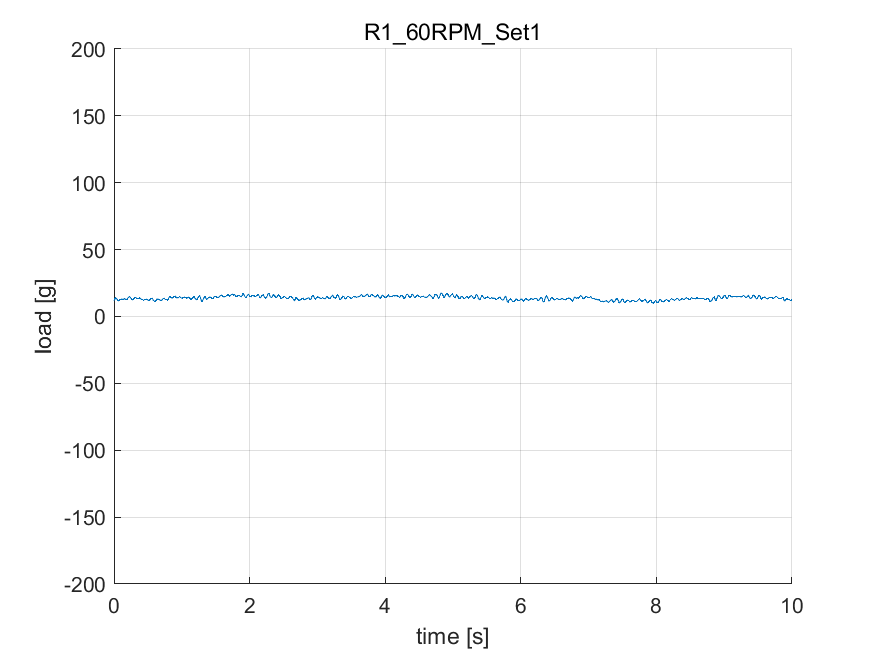

Supplement: Supplementary file 1 — Supplementary Information. [file 41598_2022_25181_MOESM1_ESM.zip › Loadcell_data_graph/R1_60RPM_Set1.png]

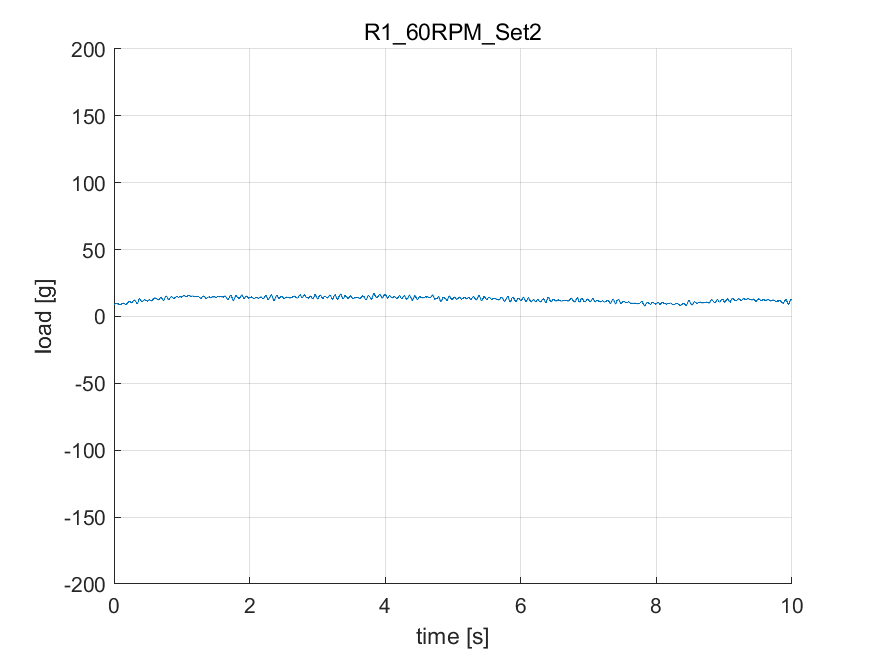

Supplement: Supplementary file 1 — Supplementary Information. [file 41598_2022_25181_MOESM1_ESM.zip › Loadcell_data_graph/R1_60RPM_Set2.png]

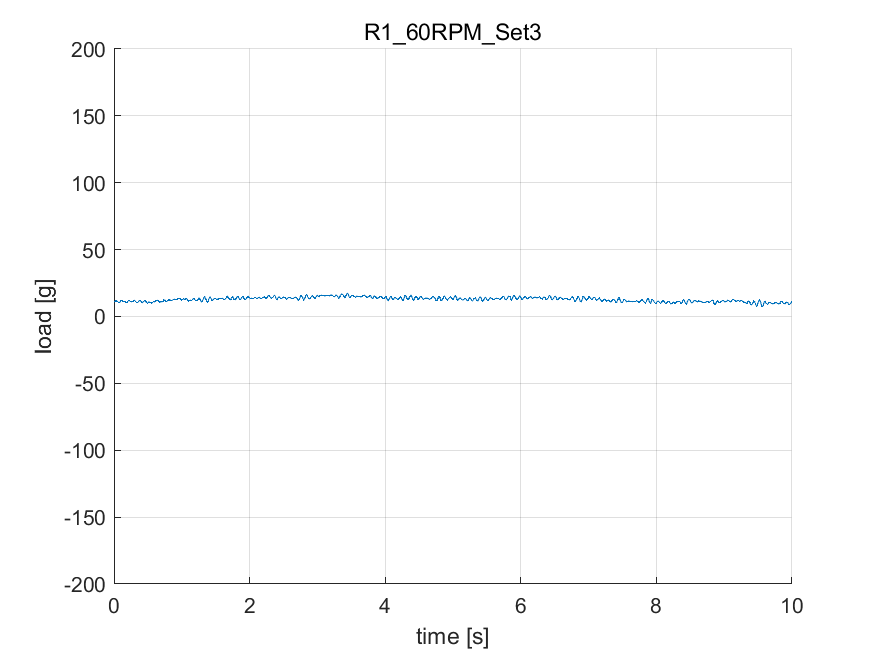

Supplement: Supplementary file 1 — Supplementary Information. [file 41598_2022_25181_MOESM1_ESM.zip › Loadcell_data_graph/R1_60RPM_Set3.png]

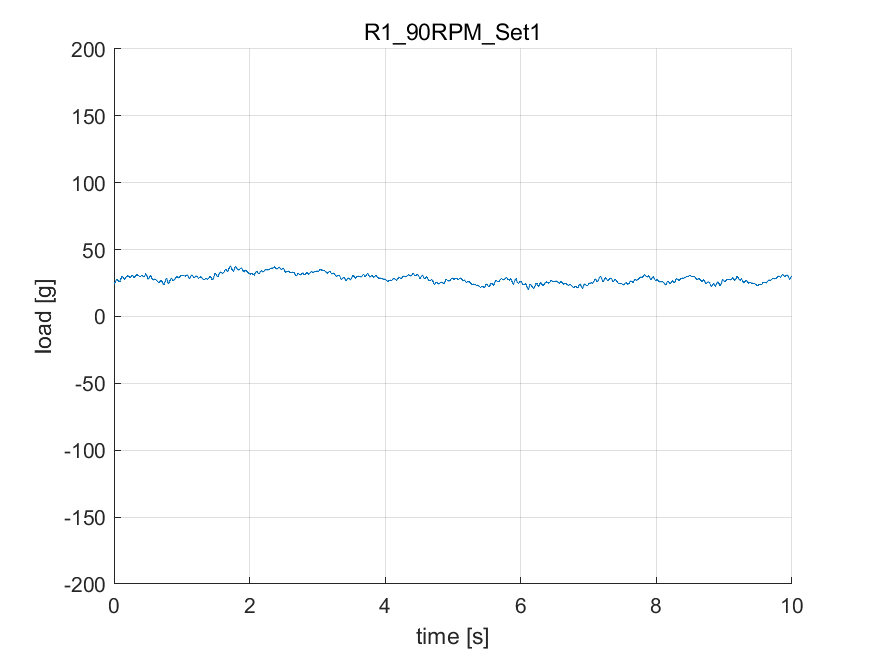

Supplement: Supplementary file 1 — Supplementary Information. [file 41598_2022_25181_MOESM1_ESM.zip › Loadcell_data_graph/R1_90RPM_Set1.png]

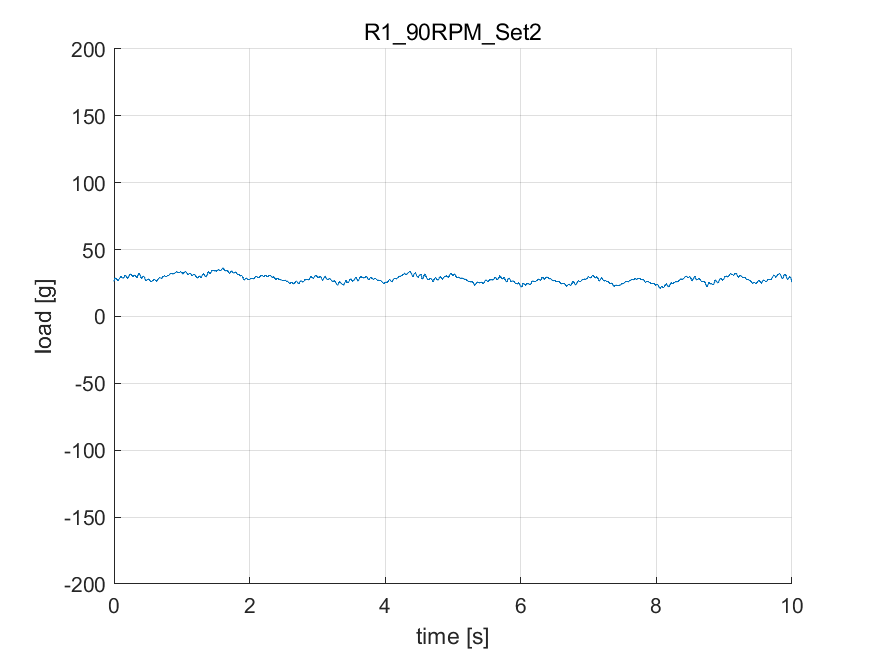

Supplement: Supplementary file 1 — Supplementary Information. [file 41598_2022_25181_MOESM1_ESM.zip › Loadcell_data_graph/R1_90RPM_Set2.png]

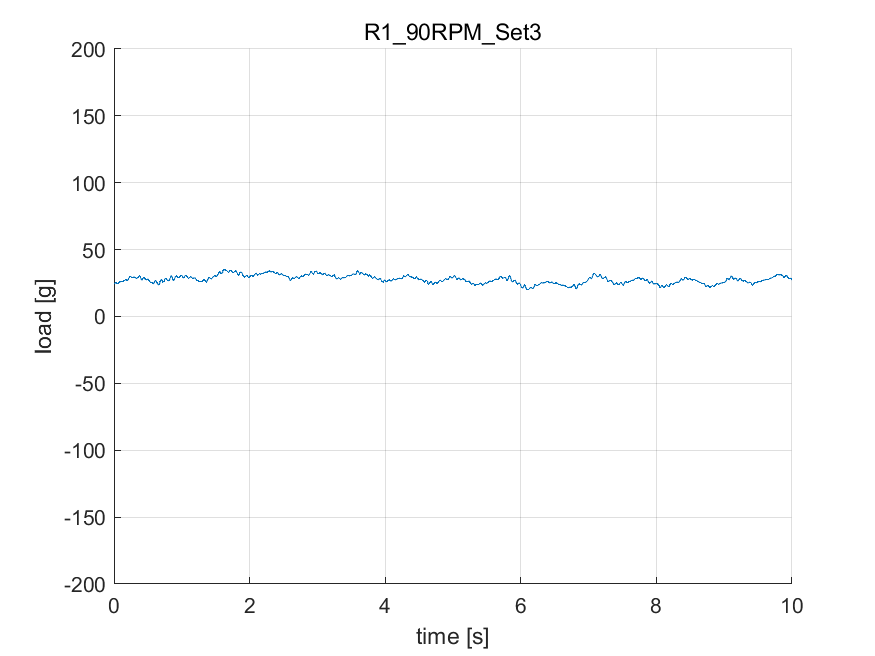

Supplement: Supplementary file 1 — Supplementary Information. [file 41598_2022_25181_MOESM1_ESM.zip › Loadcell_data_graph/R1_90RPM_Set3.png]

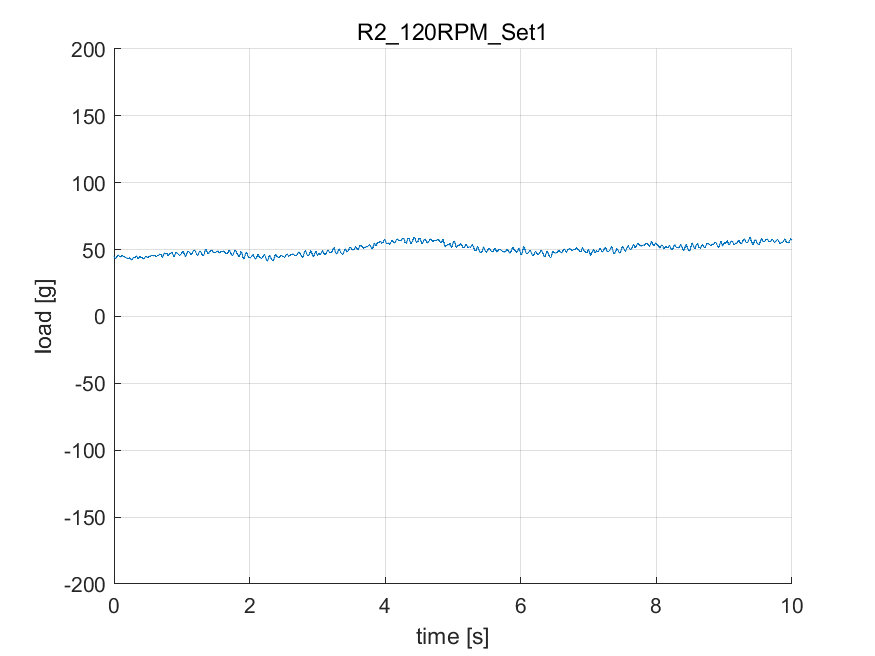

Supplement: Supplementary file 1 — Supplementary Information. [file 41598_2022_25181_MOESM1_ESM.zip › Loadcell_data_graph/R2_120RPM_Set1.png]

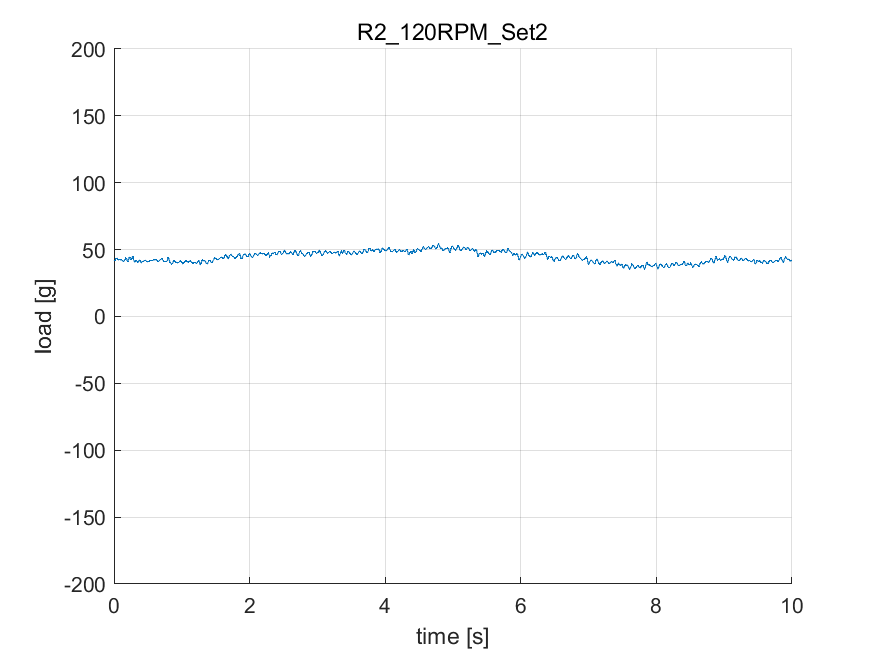

Supplement: Supplementary file 1 — Supplementary Information. [file 41598_2022_25181_MOESM1_ESM.zip › Loadcell_data_graph/R2_120RPM_Set2.png]

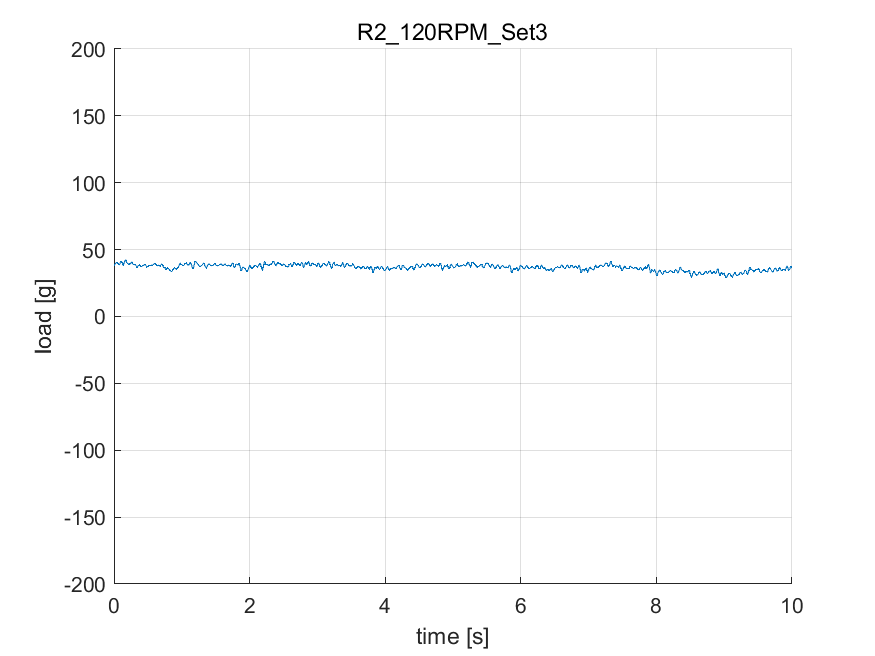

Supplement: Supplementary file 1 — Supplementary Information. [file 41598_2022_25181_MOESM1_ESM.zip › Loadcell_data_graph/R2_120RPM_Set3.png]

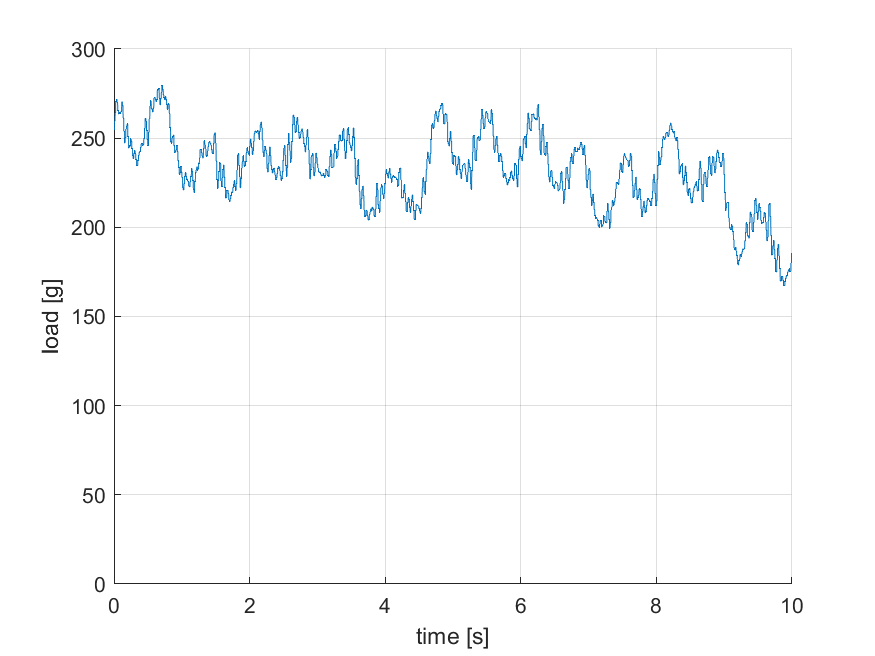

Supplement: Supplementary file 1 — Supplementary Information. [file 41598_2022_25181_MOESM1_ESM.zip › Loadcell_data_graph/R2_180RPM_Set1.png]

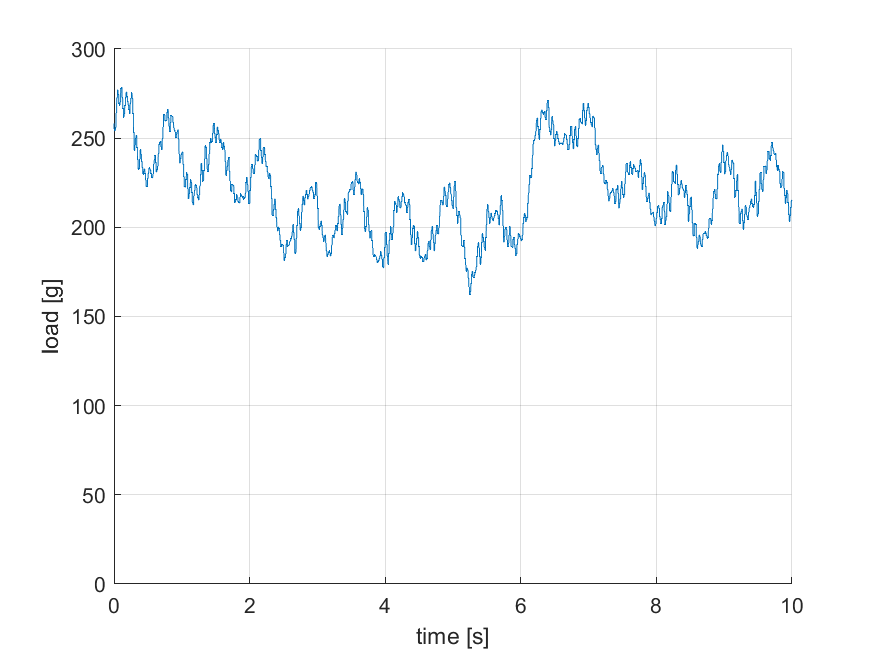

Supplement: Supplementary file 1 — Supplementary Information. [file 41598_2022_25181_MOESM1_ESM.zip › Loadcell_data_graph/R2_180RPM_Set2.png]

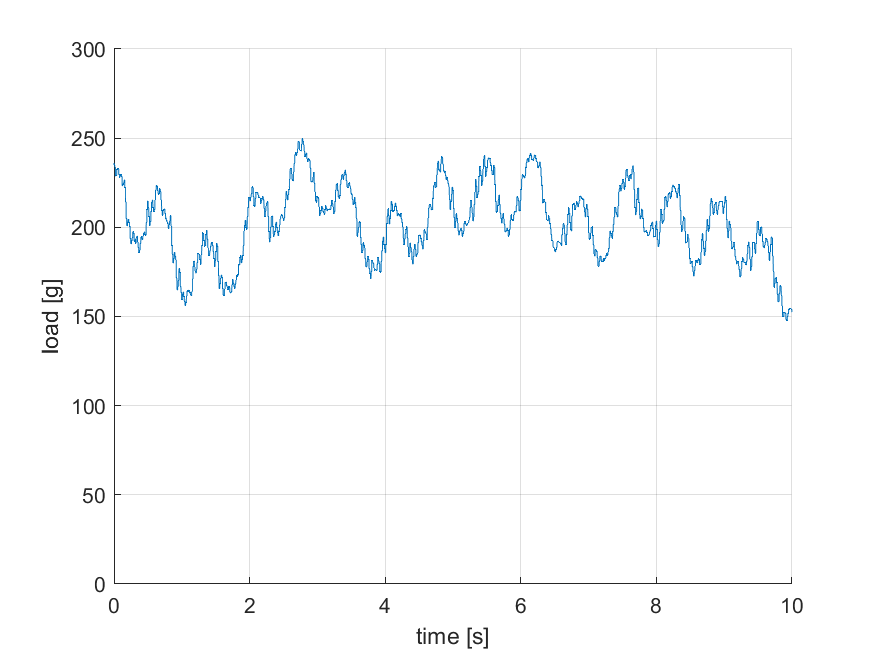

Supplement: Supplementary file 1 — Supplementary Information. [file 41598_2022_25181_MOESM1_ESM.zip › Loadcell_data_graph/R2_180RPM_Set3.png]

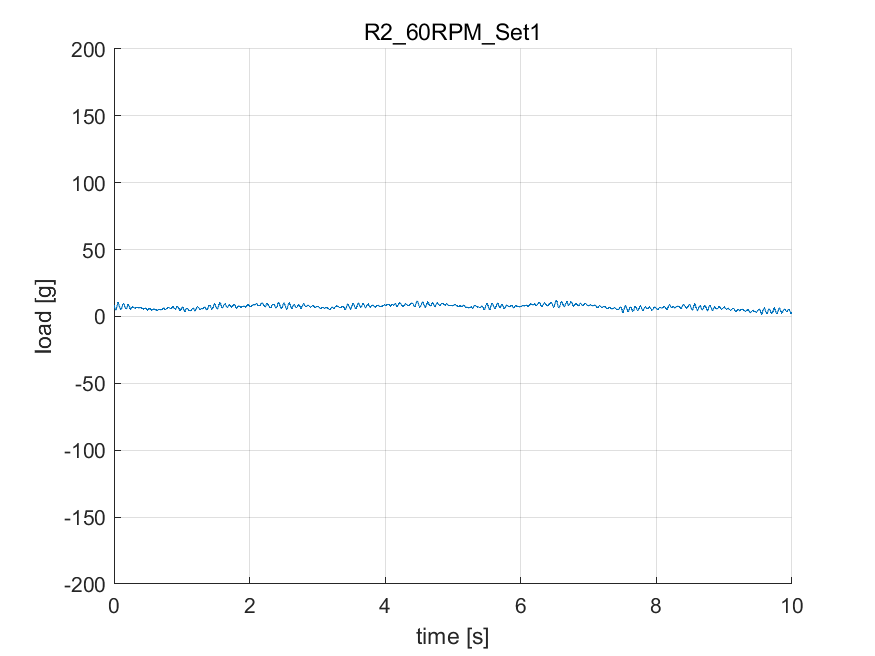

Supplement: Supplementary file 1 — Supplementary Information. [file 41598_2022_25181_MOESM1_ESM.zip › Loadcell_data_graph/R2_60RPM_Set1.png]

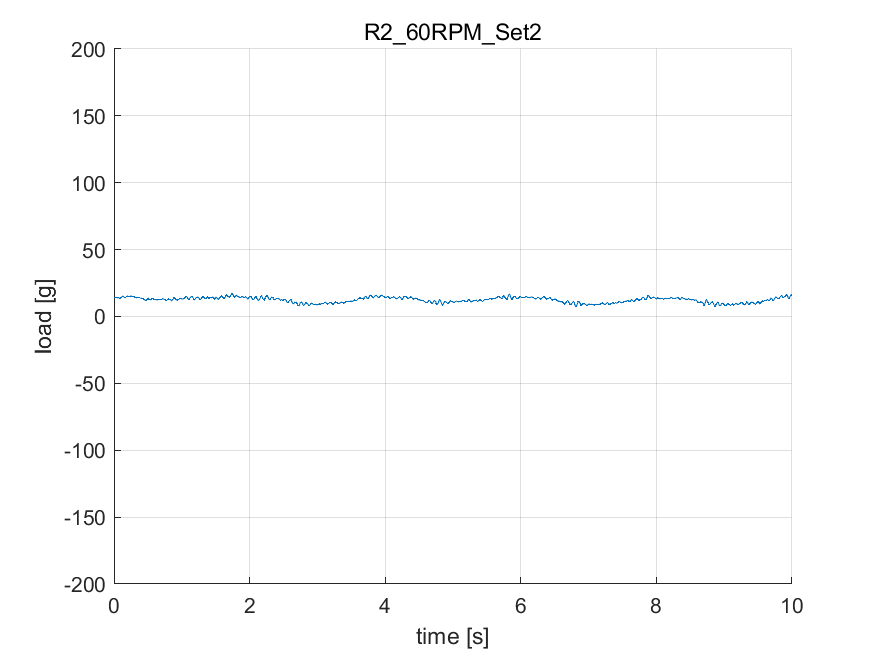

Supplement: Supplementary file 1 — Supplementary Information. [file 41598_2022_25181_MOESM1_ESM.zip › Loadcell_data_graph/R2_60RPM_Set2.png]

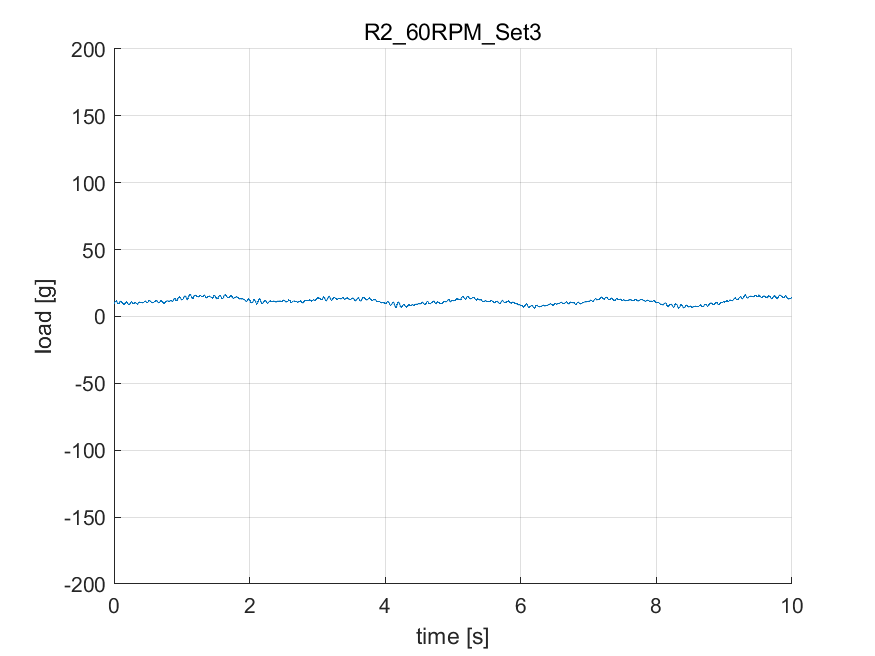

Supplement: Supplementary file 1 — Supplementary Information. [file 41598_2022_25181_MOESM1_ESM.zip › Loadcell_data_graph/R2_60RPM_Set3.png]

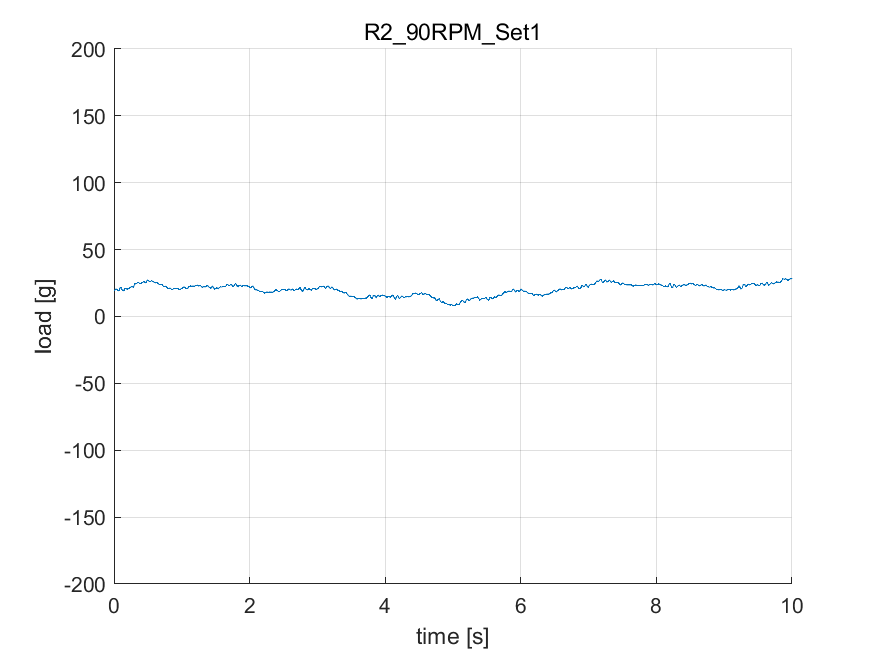

Supplement: Supplementary file 1 — Supplementary Information. [file 41598_2022_25181_MOESM1_ESM.zip › Loadcell_data_graph/R2_90RPM_Set1.png]

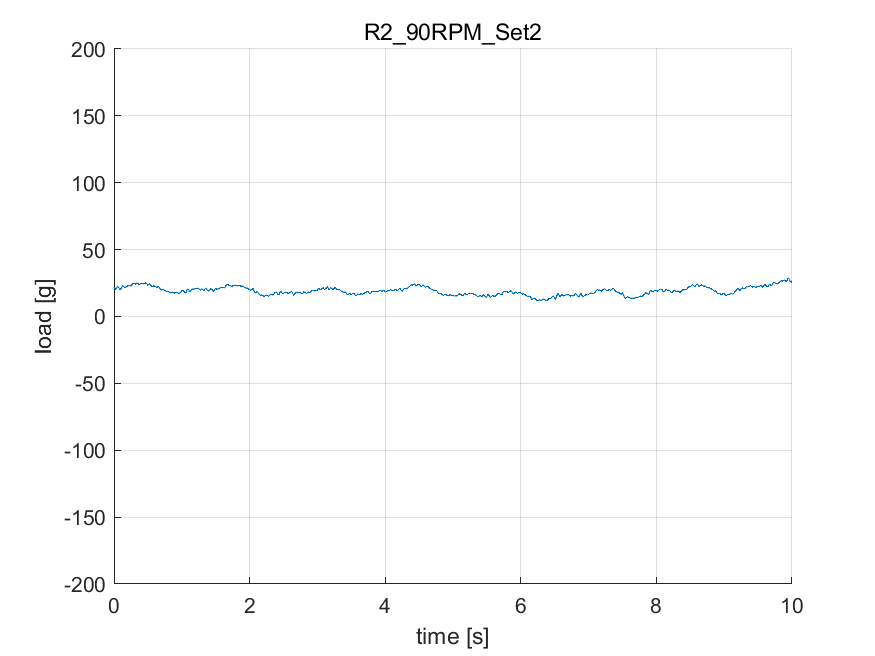

Supplement: Supplementary file 1 — Supplementary Information. [file 41598_2022_25181_MOESM1_ESM.zip › Loadcell_data_graph/R2_90RPM_Set2.png]

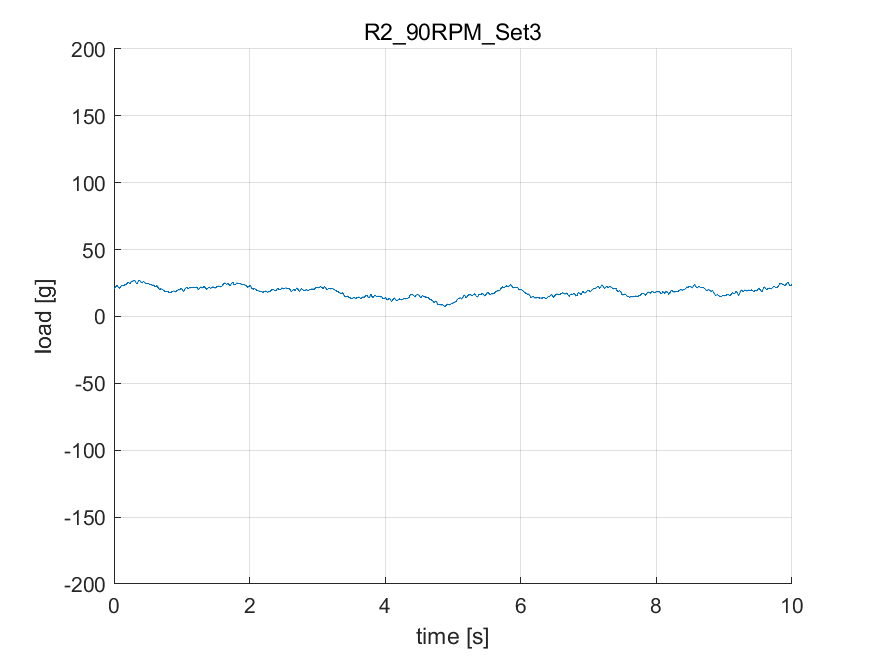

Supplement: Supplementary file 1 — Supplementary Information. [file 41598_2022_25181_MOESM1_ESM.zip › Loadcell_data_graph/R2_90RPM_Set3.png]

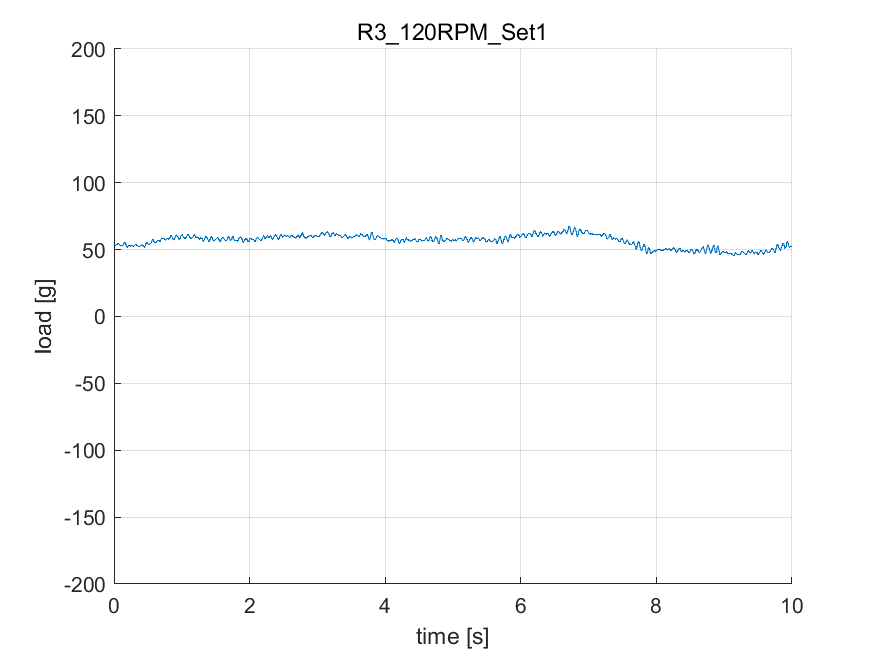

Supplement: Supplementary file 1 — Supplementary Information. [file 41598_2022_25181_MOESM1_ESM.zip › Loadcell_data_graph/R3_120RPM_Set1.png]

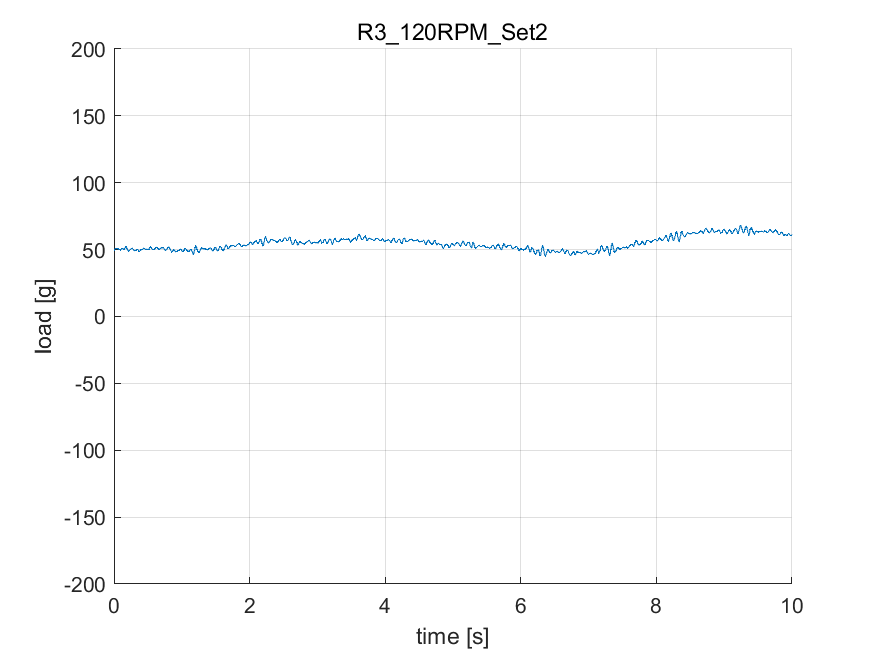

Supplement: Supplementary file 1 — Supplementary Information. [file 41598_2022_25181_MOESM1_ESM.zip › Loadcell_data_graph/R3_120RPM_Set2.png]

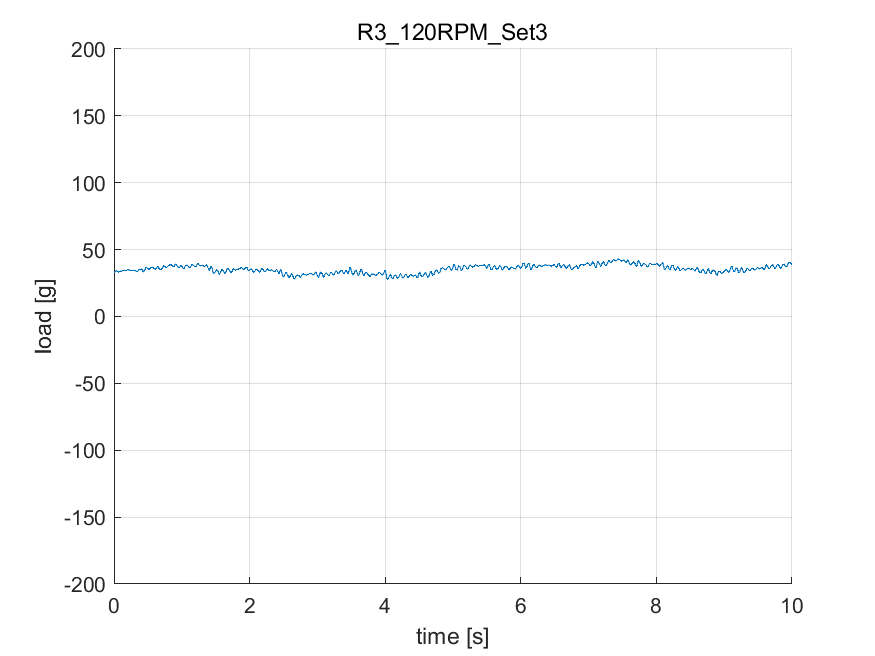

Supplement: Supplementary file 1 — Supplementary Information. [file 41598_2022_25181_MOESM1_ESM.zip › Loadcell_data_graph/R3_120RPM_Set3.png]

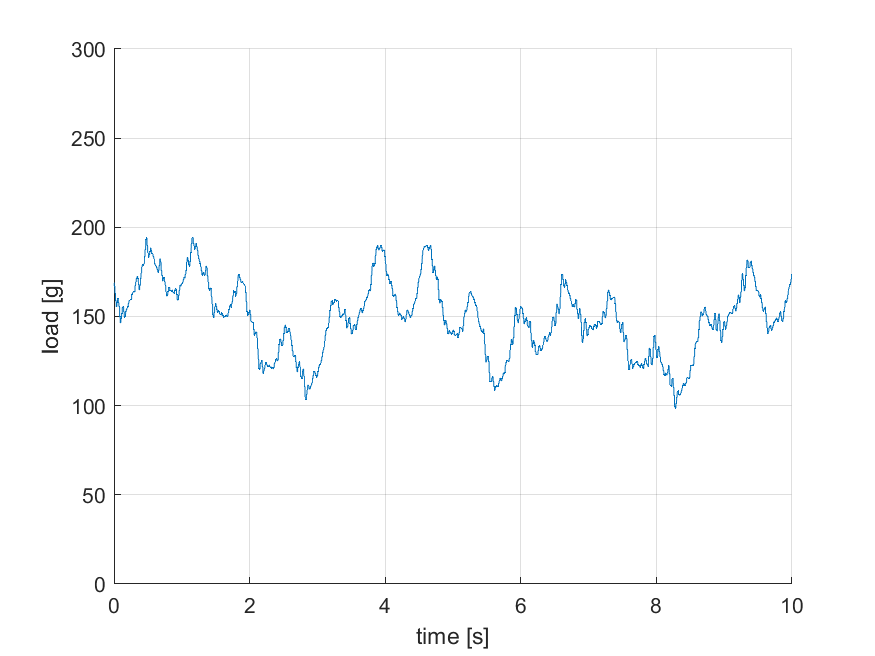

Supplement: Supplementary file 1 — Supplementary Information. [file 41598_2022_25181_MOESM1_ESM.zip › Loadcell_data_graph/R3_180RPM_Set1.png]

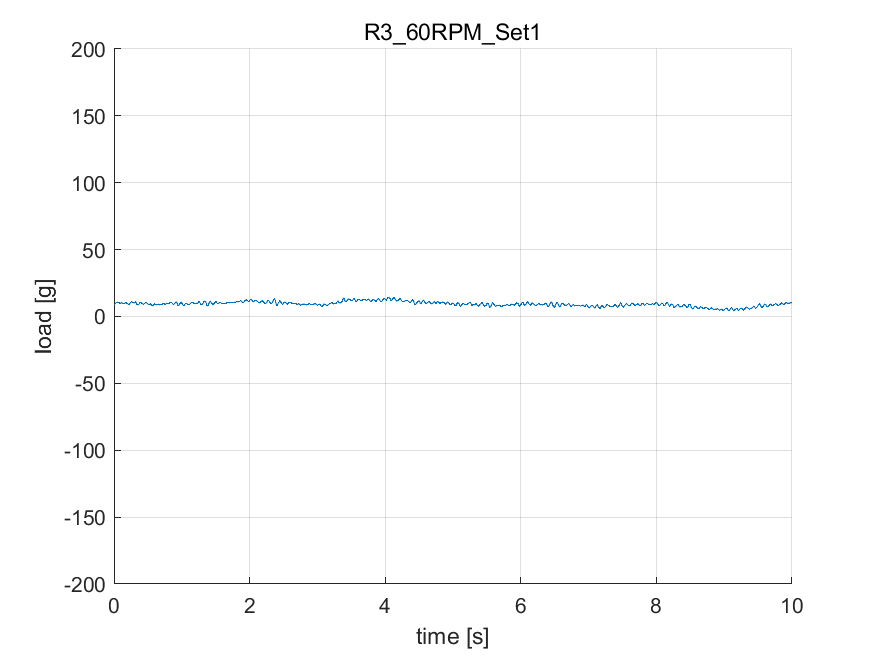

Supplement: Supplementary file 1 — Supplementary Information. [file 41598_2022_25181_MOESM1_ESM.zip › Loadcell_data_graph/R3_60RPM_Set1.png]

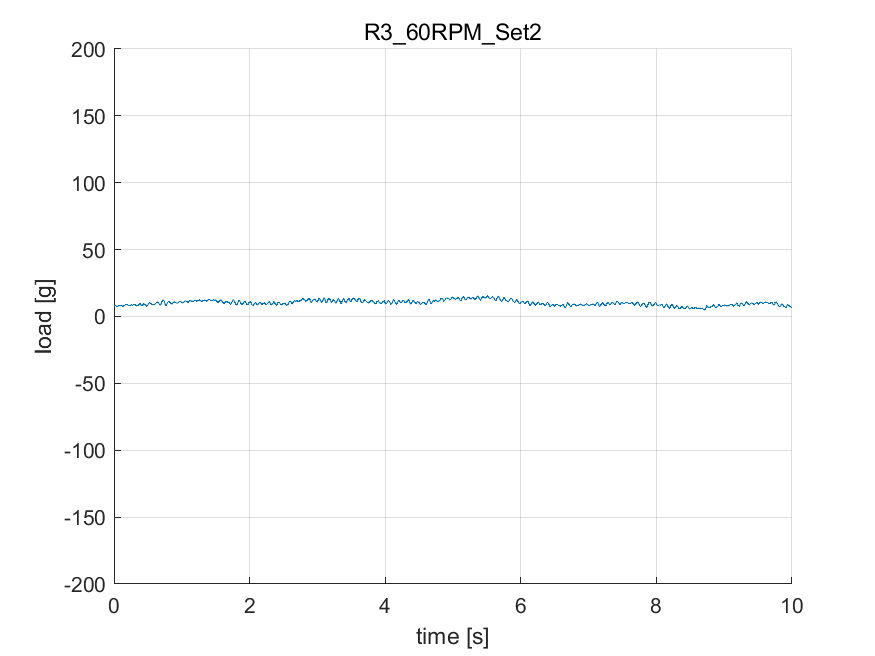

Supplement: Supplementary file 1 — Supplementary Information. [file 41598_2022_25181_MOESM1_ESM.zip › Loadcell_data_graph/R3_60RPM_Set2.png]

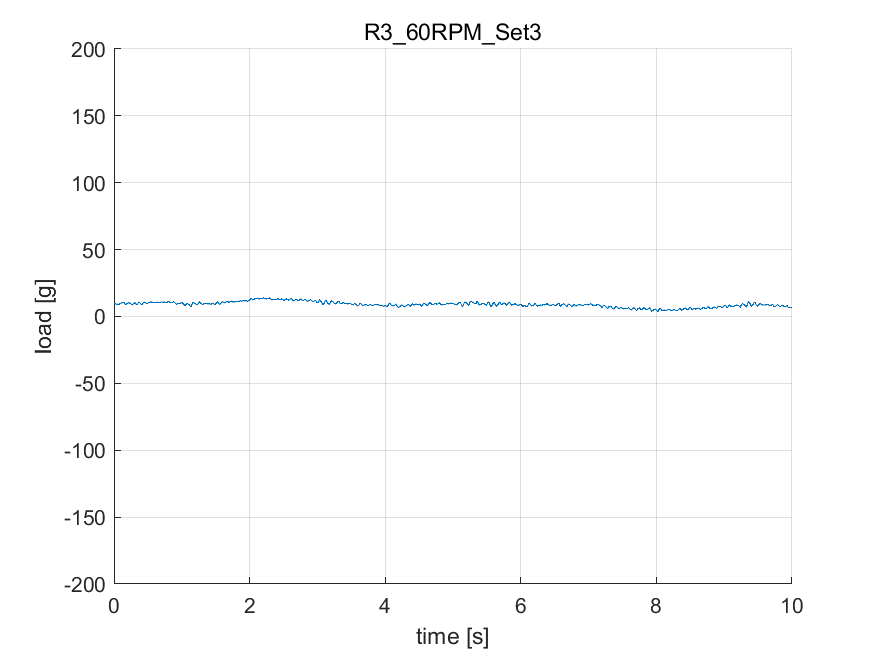

Supplement: Supplementary file 1 — Supplementary Information. [file 41598_2022_25181_MOESM1_ESM.zip › Loadcell_data_graph/R3_60RPM_Set3.png]

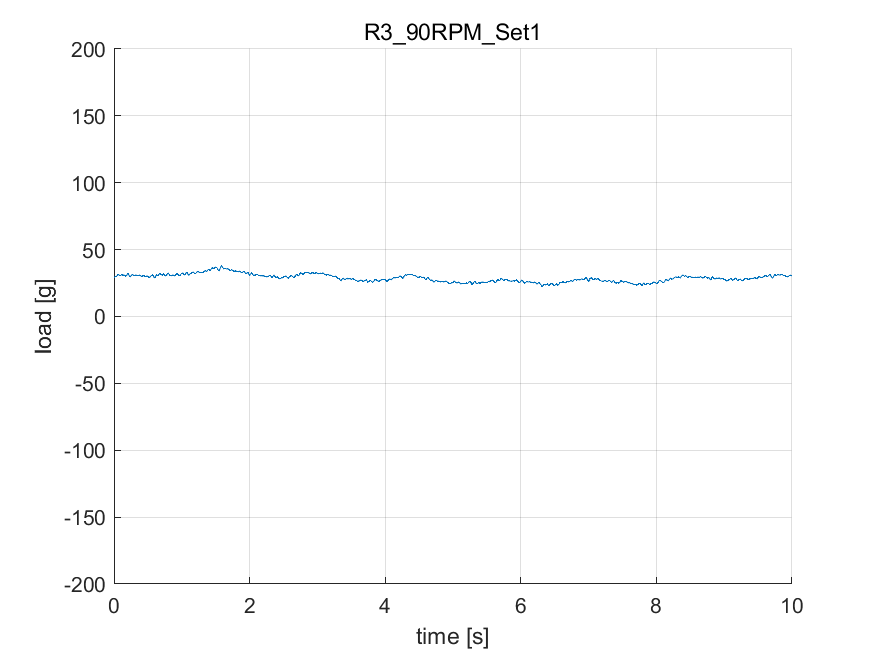

Supplement: Supplementary file 1 — Supplementary Information. [file 41598_2022_25181_MOESM1_ESM.zip › Loadcell_data_graph/R3_90RPM_Set1.png]

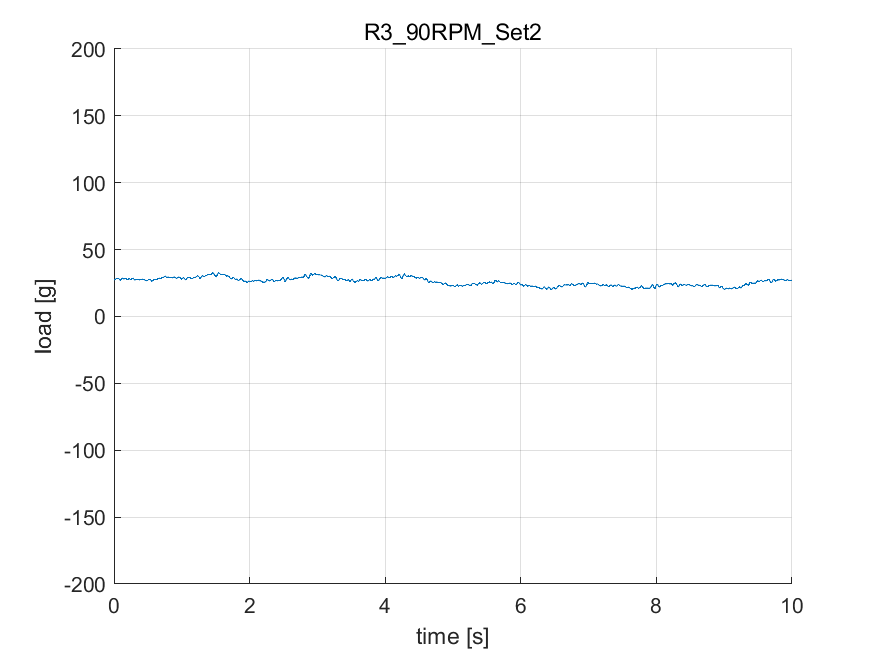

Supplement: Supplementary file 1 — Supplementary Information. [file 41598_2022_25181_MOESM1_ESM.zip › Loadcell_data_graph/R3_90RPM_Set2.png]

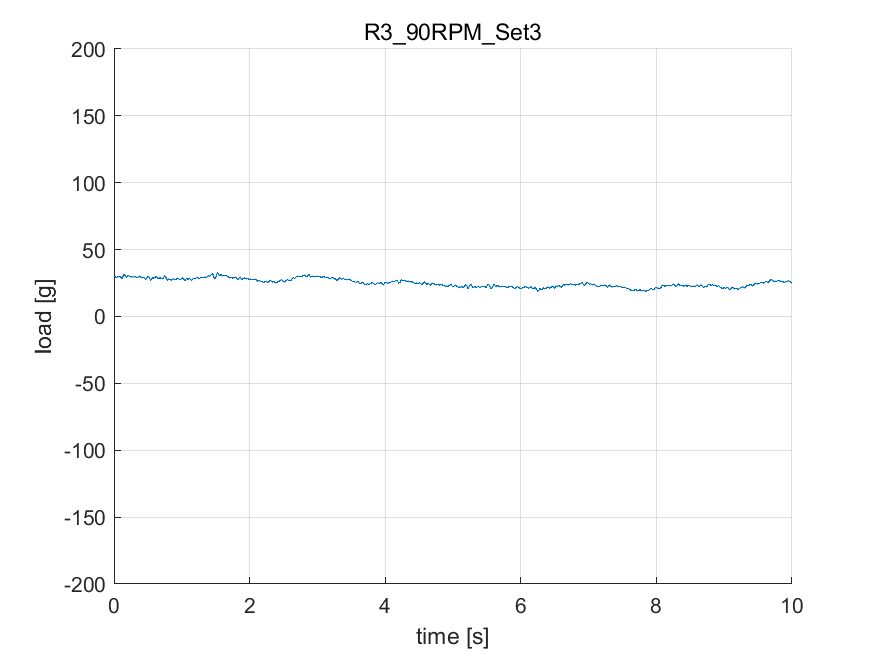

Supplement: Supplementary file 1 — Supplementary Information. [file 41598_2022_25181_MOESM1_ESM.zip › Loadcell_data_graph/R3_90RPM_Set3.png]

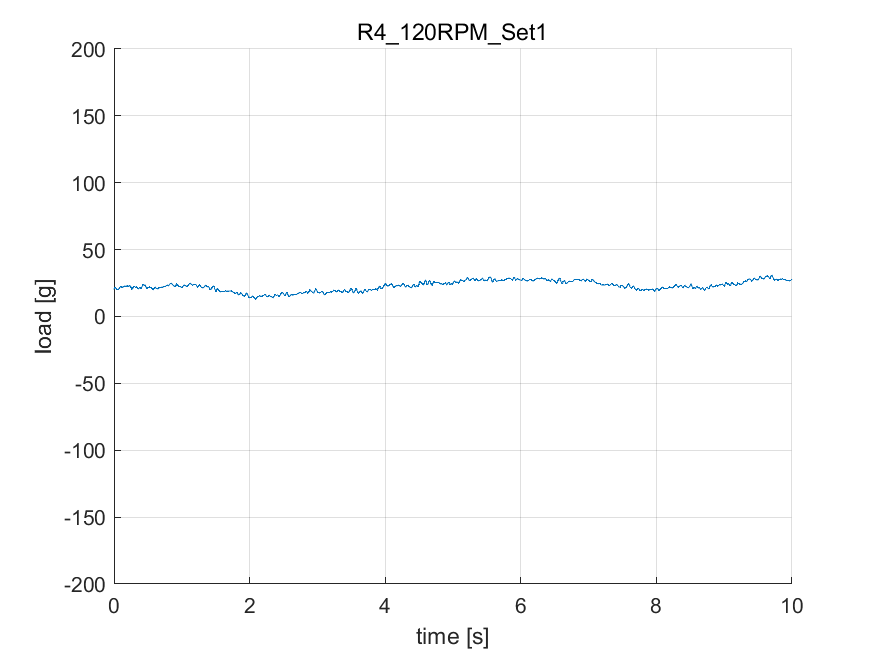

Supplement: Supplementary file 1 — Supplementary Information. [file 41598_2022_25181_MOESM1_ESM.zip › Loadcell_data_graph/R4_120RPM_Set1.png]

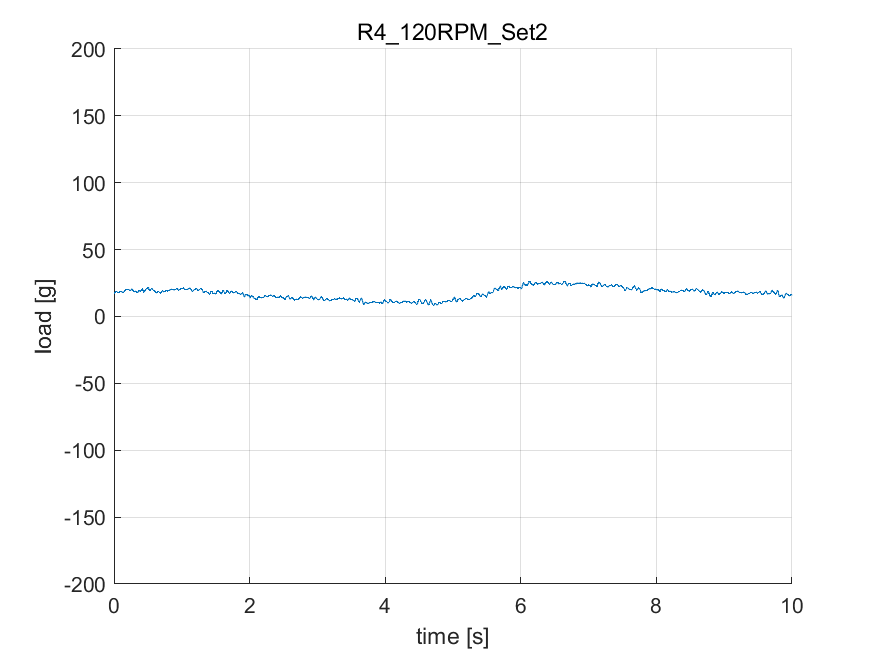

Supplement: Supplementary file 1 — Supplementary Information. [file 41598_2022_25181_MOESM1_ESM.zip › Loadcell_data_graph/R4_120RPM_Set2.png]

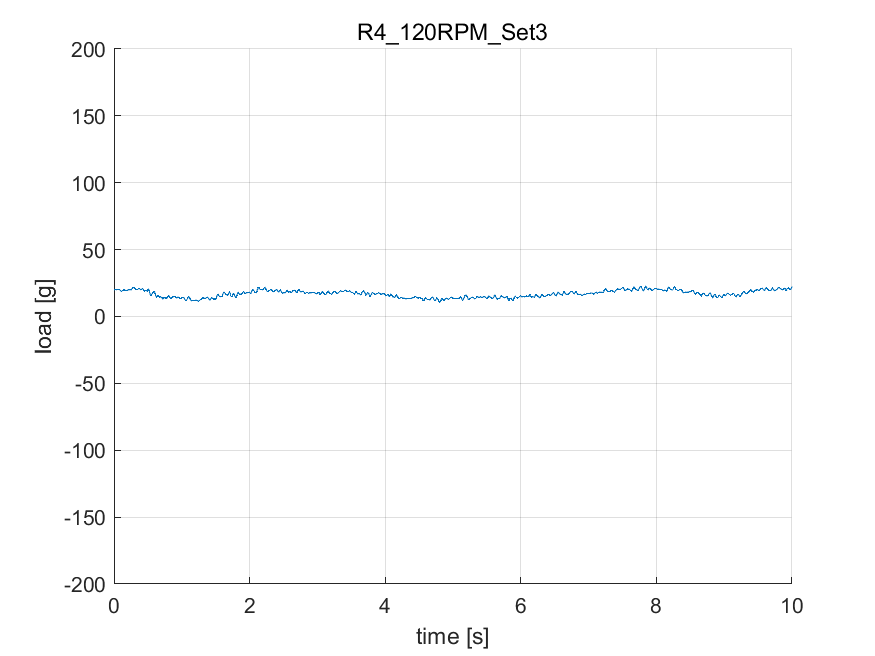

Supplement: Supplementary file 1 — Supplementary Information. [file 41598_2022_25181_MOESM1_ESM.zip › Loadcell_data_graph/R4_120RPM_Set3.png]

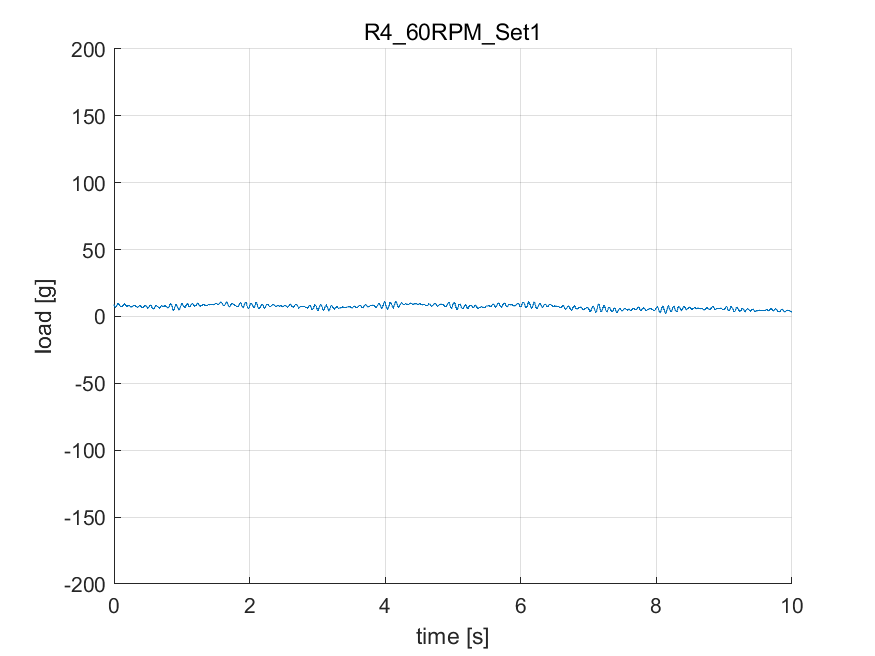

Supplement: Supplementary file 1 — Supplementary Information. [file 41598_2022_25181_MOESM1_ESM.zip › Loadcell_data_graph/R4_60RPM_Set1.png]

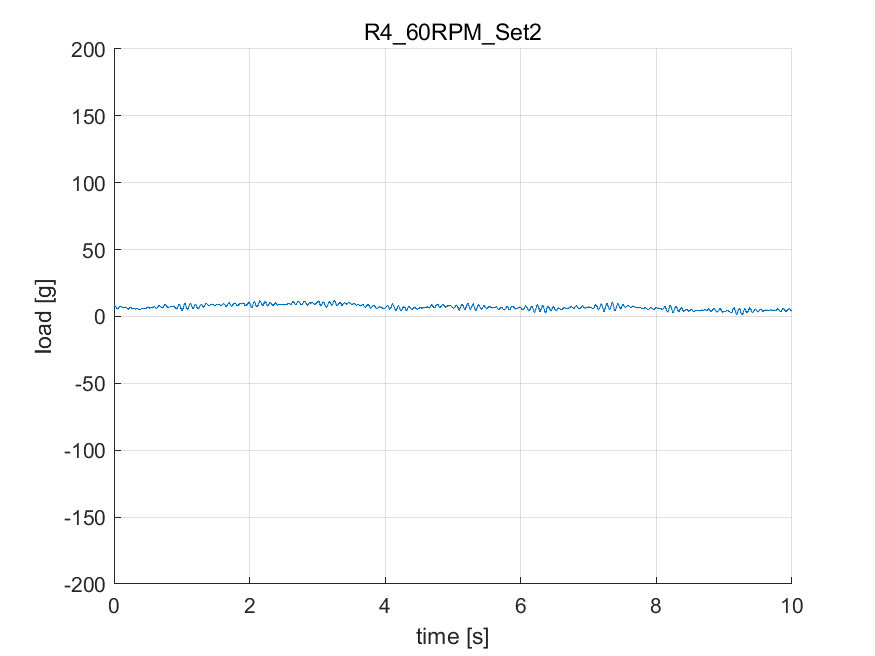

Supplement: Supplementary file 1 — Supplementary Information. [file 41598_2022_25181_MOESM1_ESM.zip › Loadcell_data_graph/R4_60RPM_Set2.png]

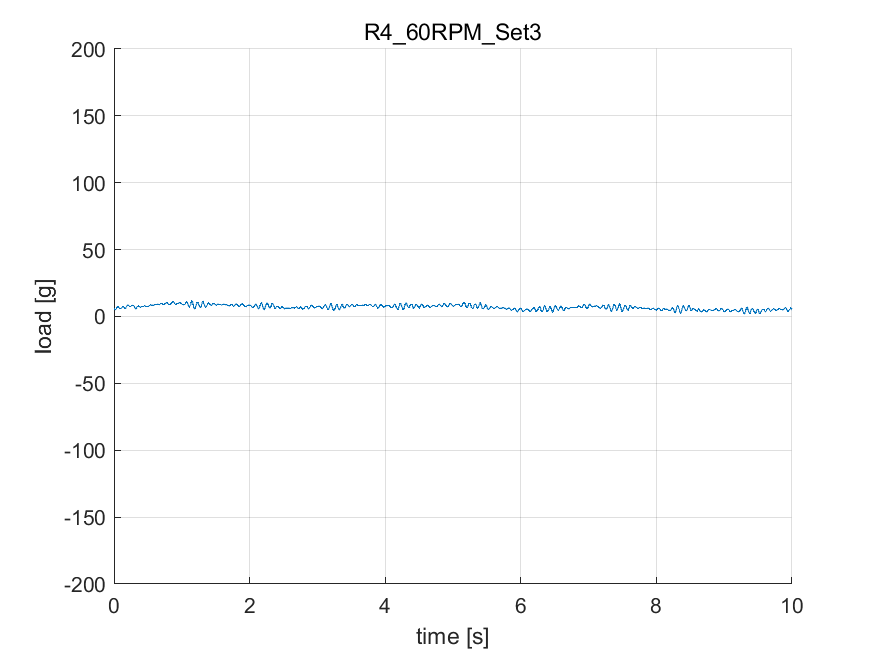

Supplement: Supplementary file 1 — Supplementary Information. [file 41598_2022_25181_MOESM1_ESM.zip › Loadcell_data_graph/R4_60RPM_Set3.png]

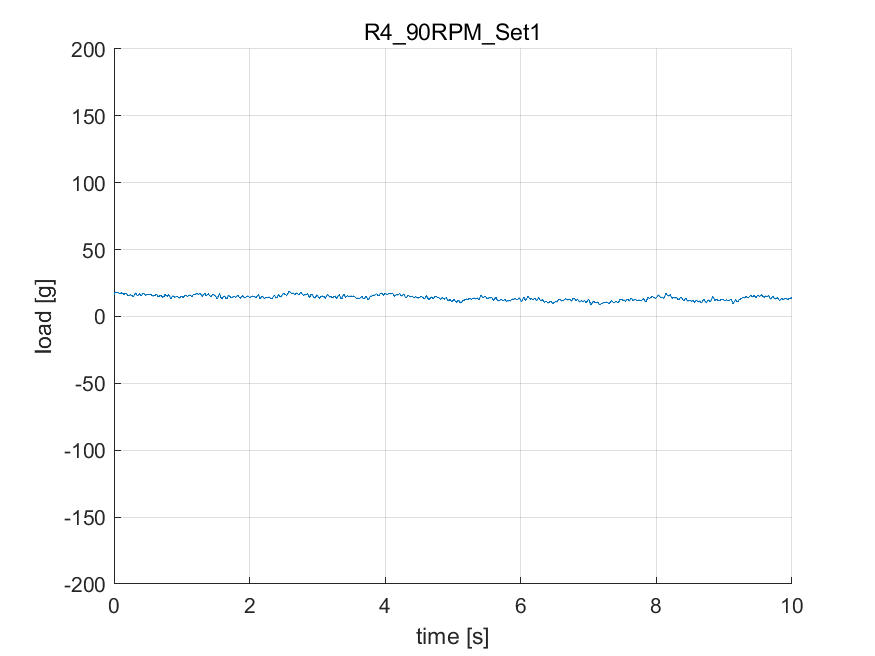

Supplement: Supplementary file 1 — Supplementary Information. [file 41598_2022_25181_MOESM1_ESM.zip › Loadcell_data_graph/R4_90RPM_Set1.png]

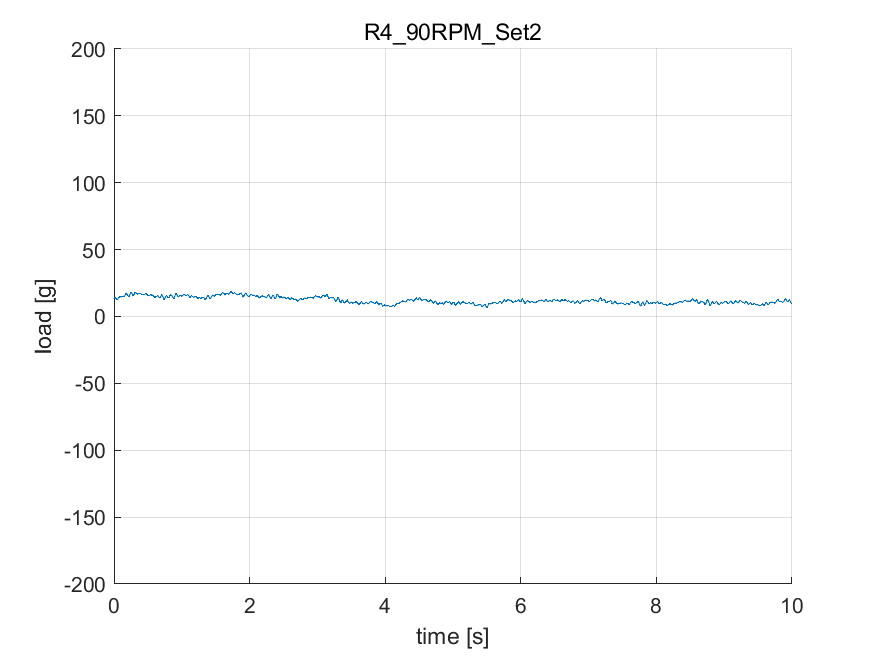

Supplement: Supplementary file 1 — Supplementary Information. [file 41598_2022_25181_MOESM1_ESM.zip › Loadcell_data_graph/R4_90RPM_Set2.png]

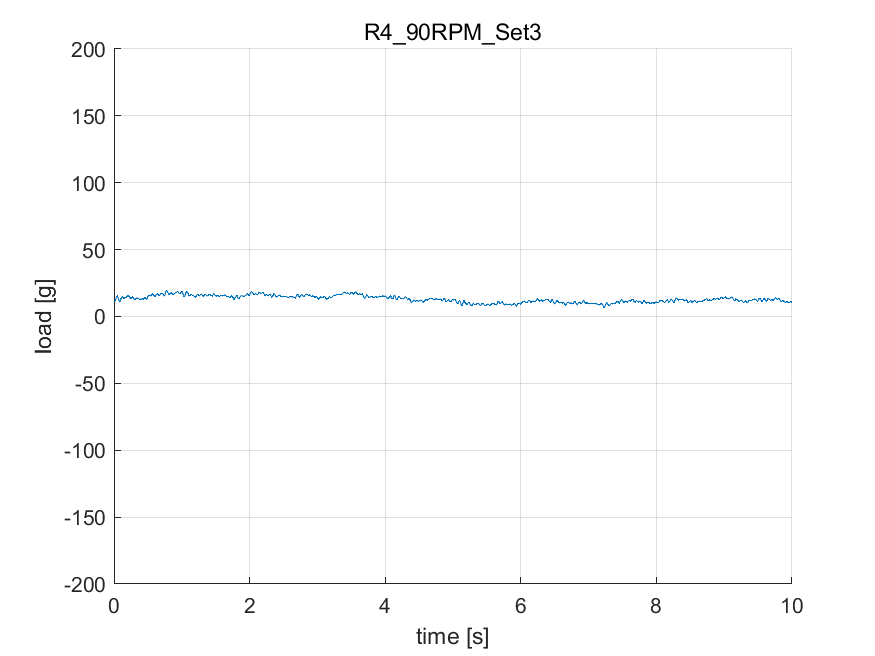

Supplement: Supplementary file 1 — Supplementary Information. [file 41598_2022_25181_MOESM1_ESM.zip › Loadcell_data_graph/R4_90RPM_Set3.png]

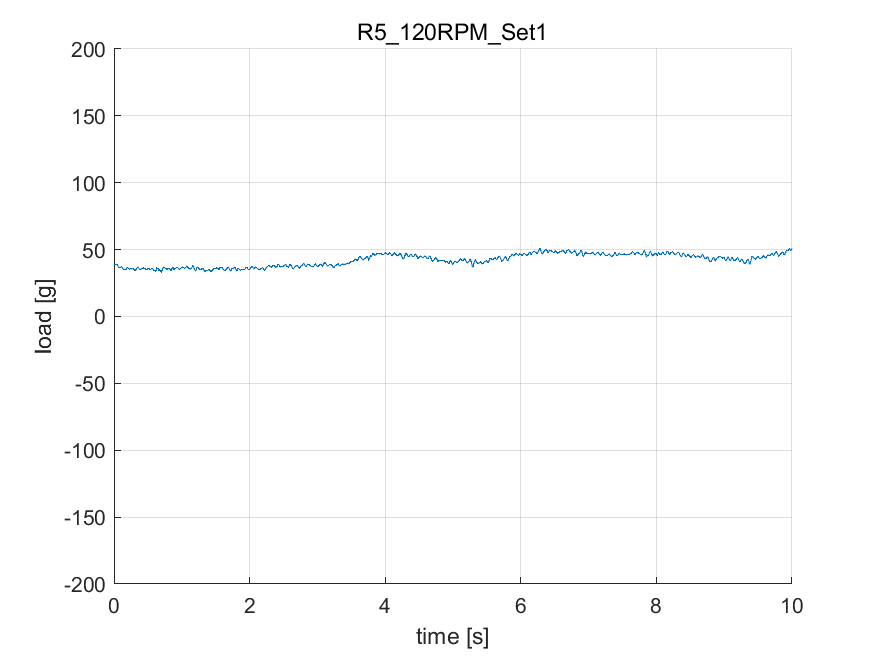

Supplement: Supplementary file 1 — Supplementary Information. [file 41598_2022_25181_MOESM1_ESM.zip › Loadcell_data_graph/R5_120RPM_Set1.png]

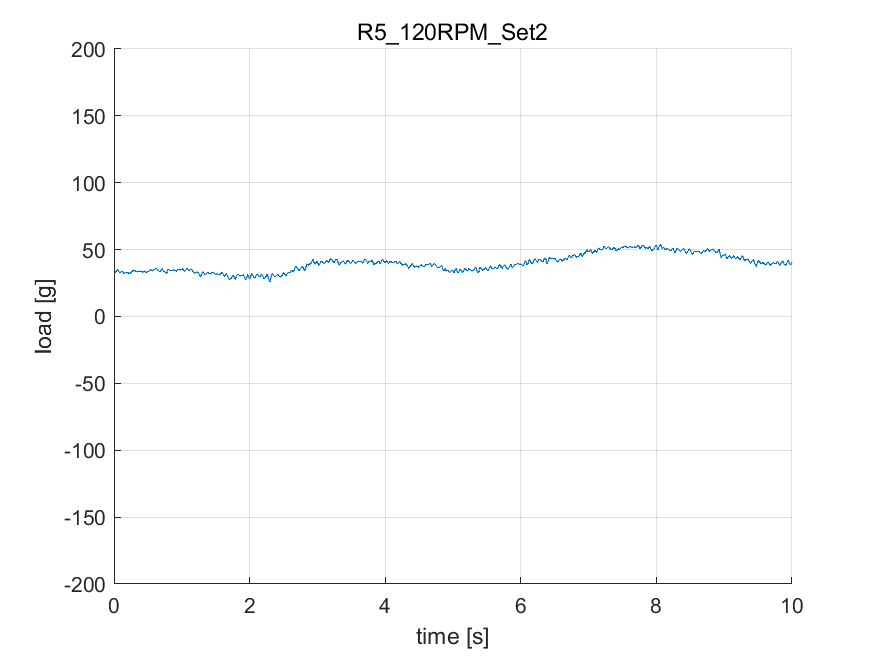

Supplement: Supplementary file 1 — Supplementary Information. [file 41598_2022_25181_MOESM1_ESM.zip › Loadcell_data_graph/R5_120RPM_Set2.png]

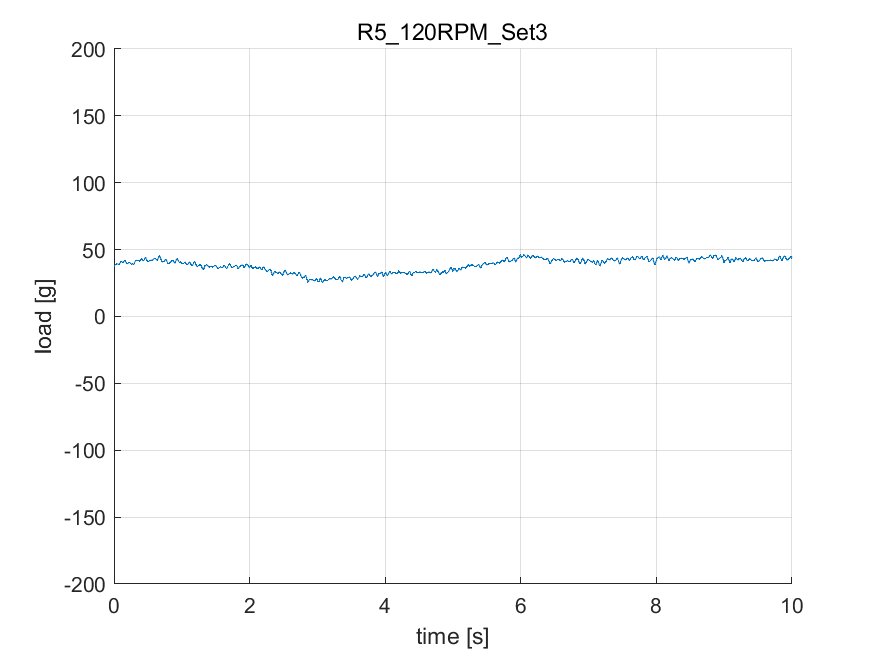

Supplement: Supplementary file 1 — Supplementary Information. [file 41598_2022_25181_MOESM1_ESM.zip › Loadcell_data_graph/R5_120RPM_Set3.png]

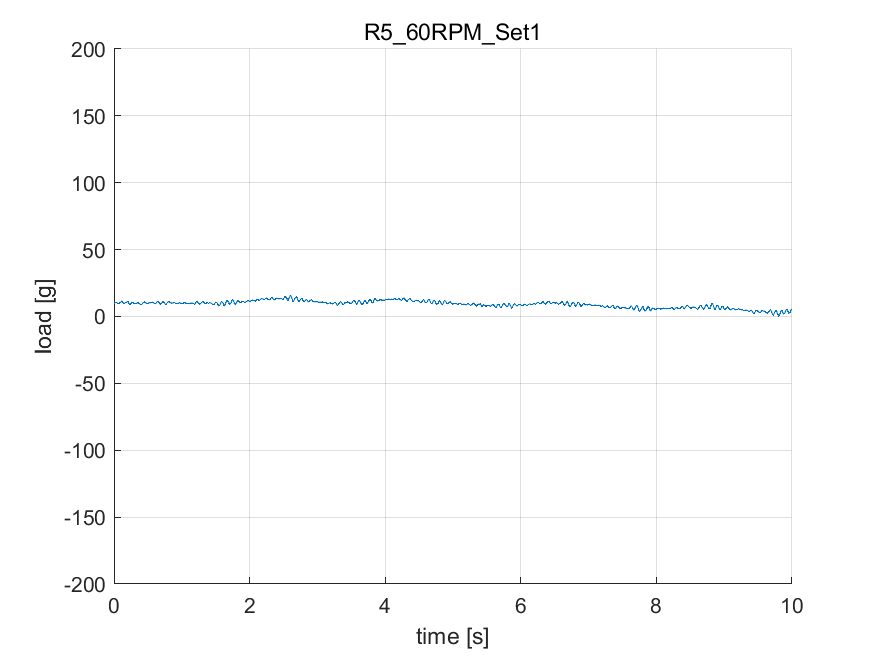

Supplement: Supplementary file 1 — Supplementary Information. [file 41598_2022_25181_MOESM1_ESM.zip › Loadcell_data_graph/R5_60RPM_Set1.png]

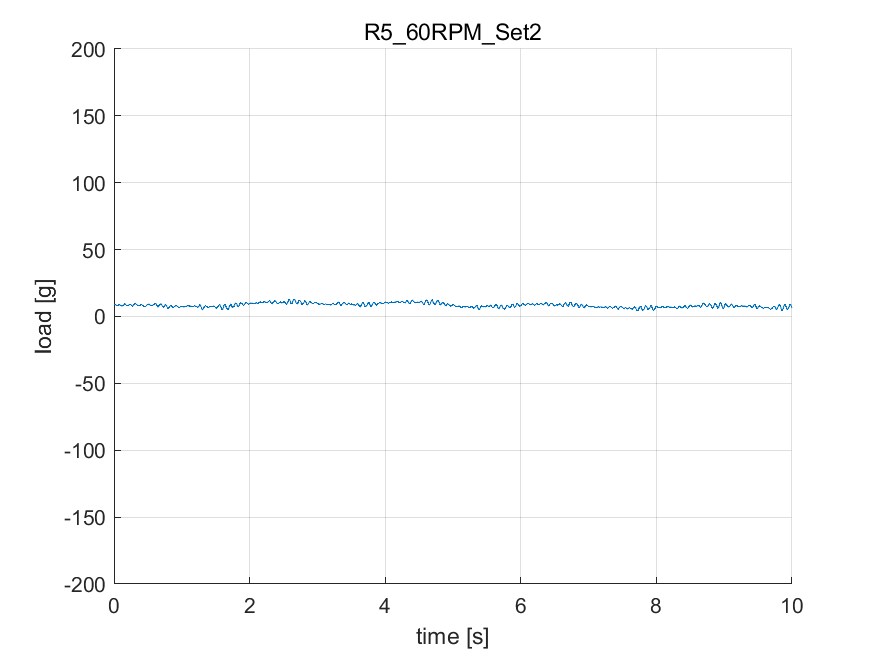

Supplement: Supplementary file 1 — Supplementary Information. [file 41598_2022_25181_MOESM1_ESM.zip › Loadcell_data_graph/R5_60RPM_Set2.png]

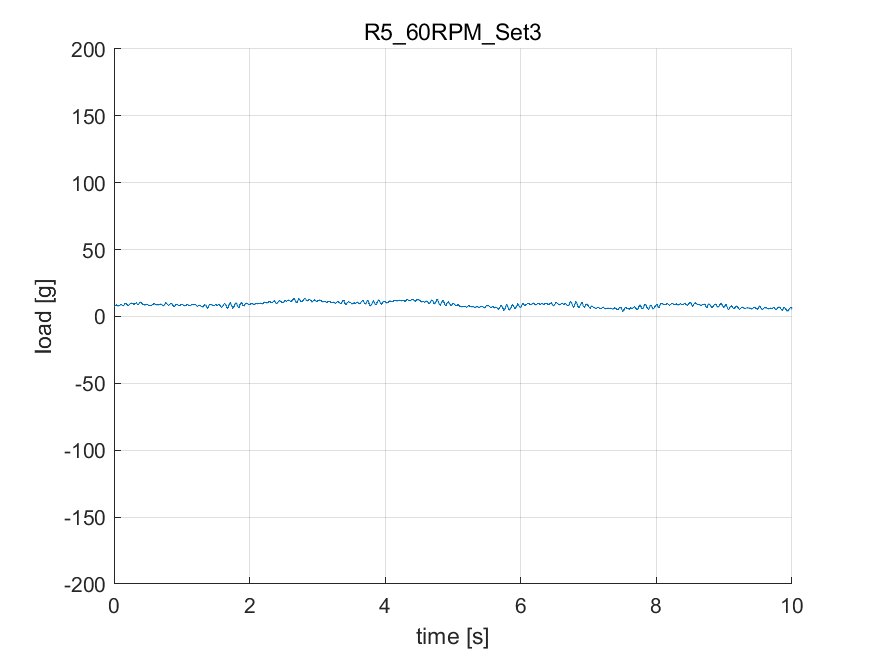

Supplement: Supplementary file 1 — Supplementary Information. [file 41598_2022_25181_MOESM1_ESM.zip › Loadcell_data_graph/R5_60RPM_Set3.png]

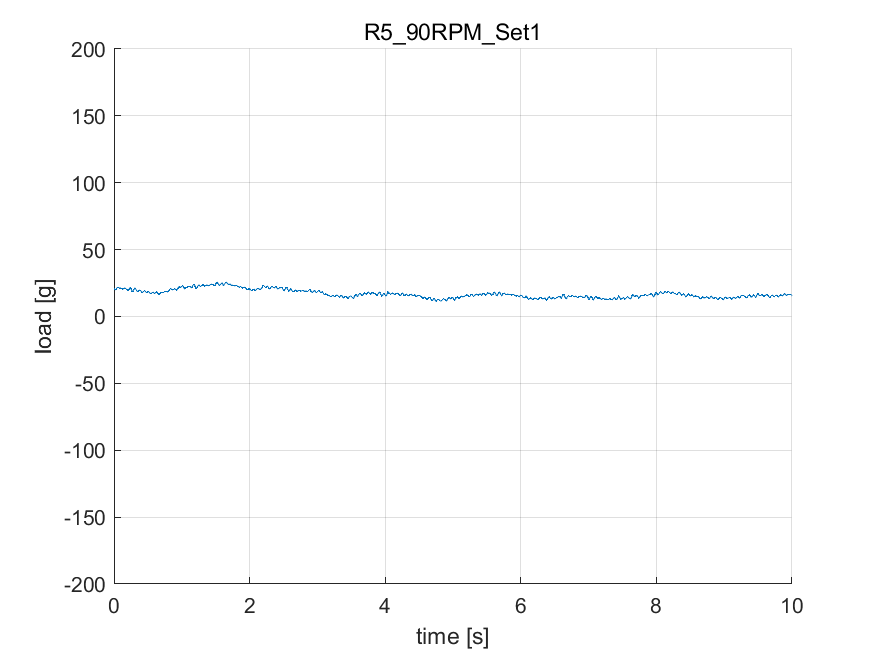

Supplement: Supplementary file 1 — Supplementary Information. [file 41598_2022_25181_MOESM1_ESM.zip › Loadcell_data_graph/R5_90RPM_Set1.png]

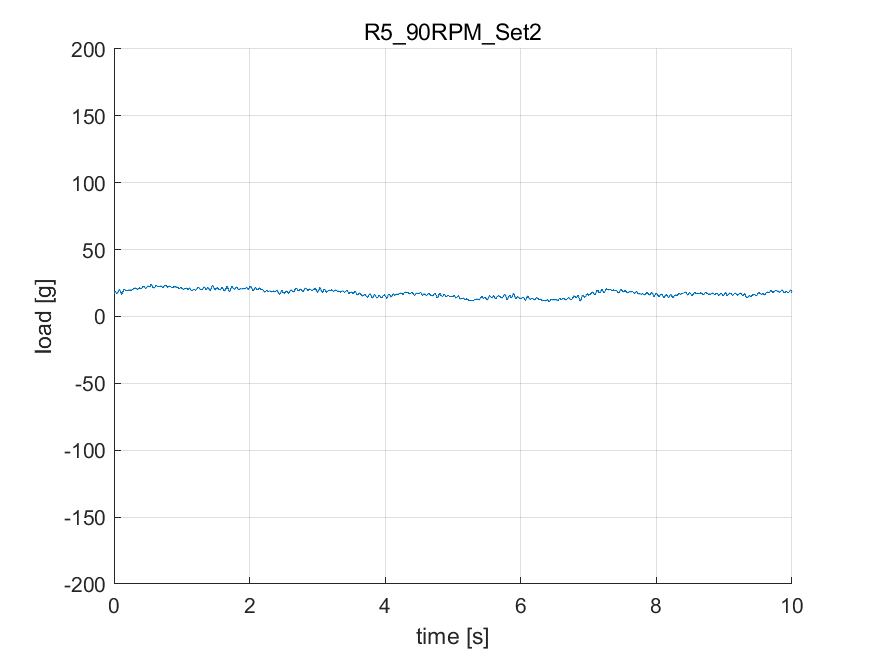

Supplement: Supplementary file 1 — Supplementary Information. [file 41598_2022_25181_MOESM1_ESM.zip › Loadcell_data_graph/R5_90RPM_Set2.png]

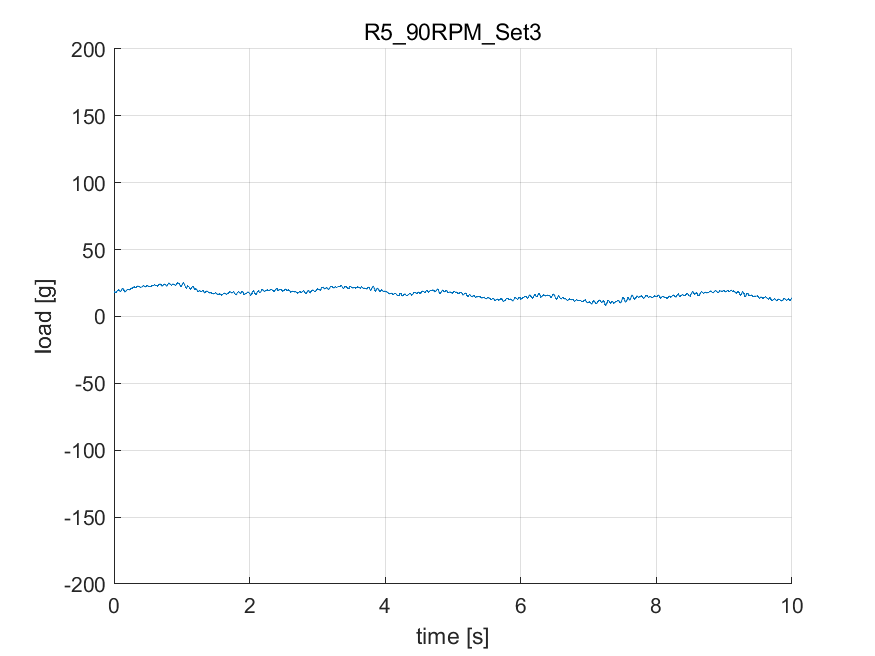

Supplement: Supplementary file 1 — Supplementary Information. [file 41598_2022_25181_MOESM1_ESM.zip › Loadcell_data_graph/R5_90RPM_Set3.png]

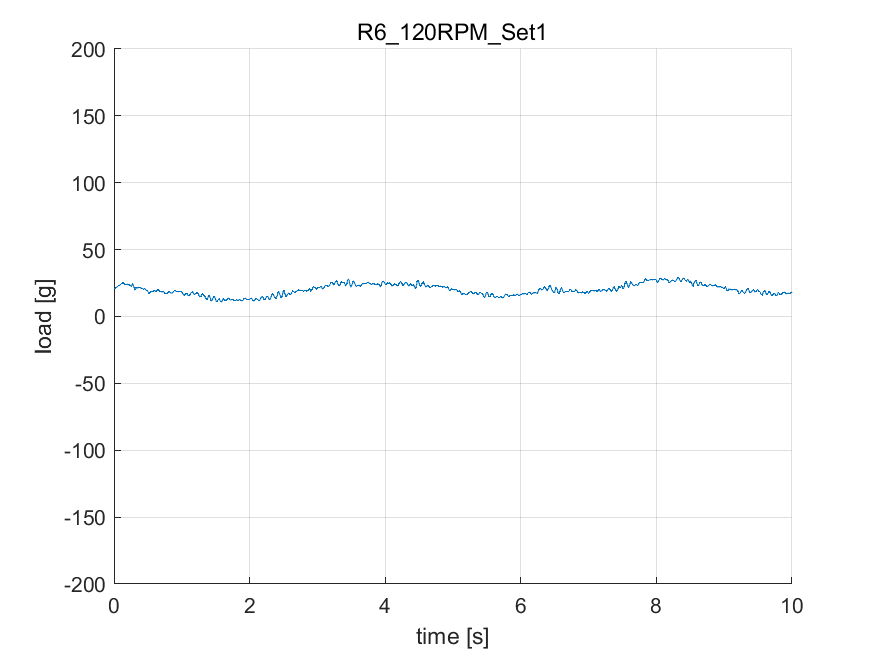

Supplement: Supplementary file 1 — Supplementary Information. [file 41598_2022_25181_MOESM1_ESM.zip › Loadcell_data_graph/R6_120RPM_Set1.png]

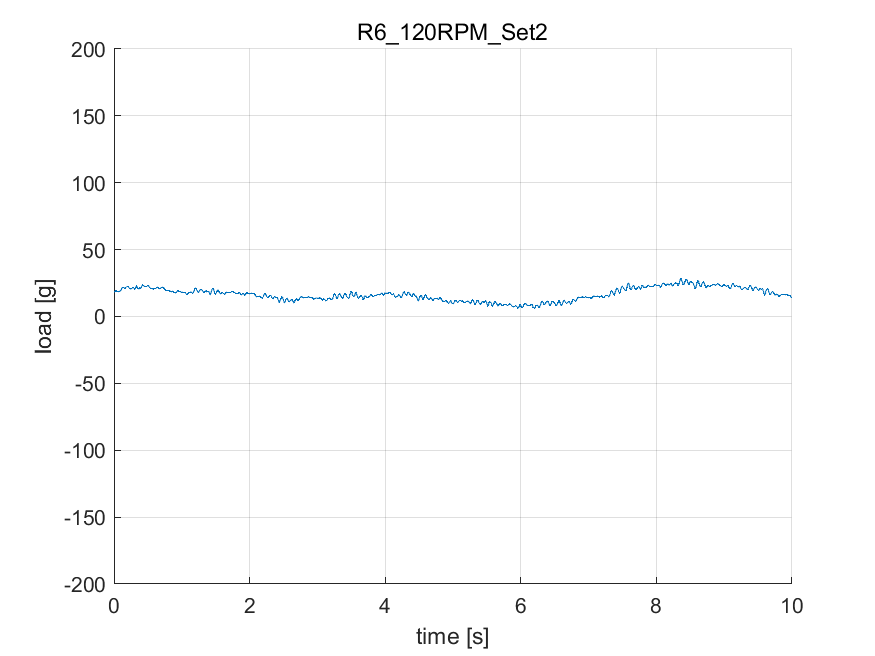

Supplement: Supplementary file 1 — Supplementary Information. [file 41598_2022_25181_MOESM1_ESM.zip › Loadcell_data_graph/R6_120RPM_Set2.png]

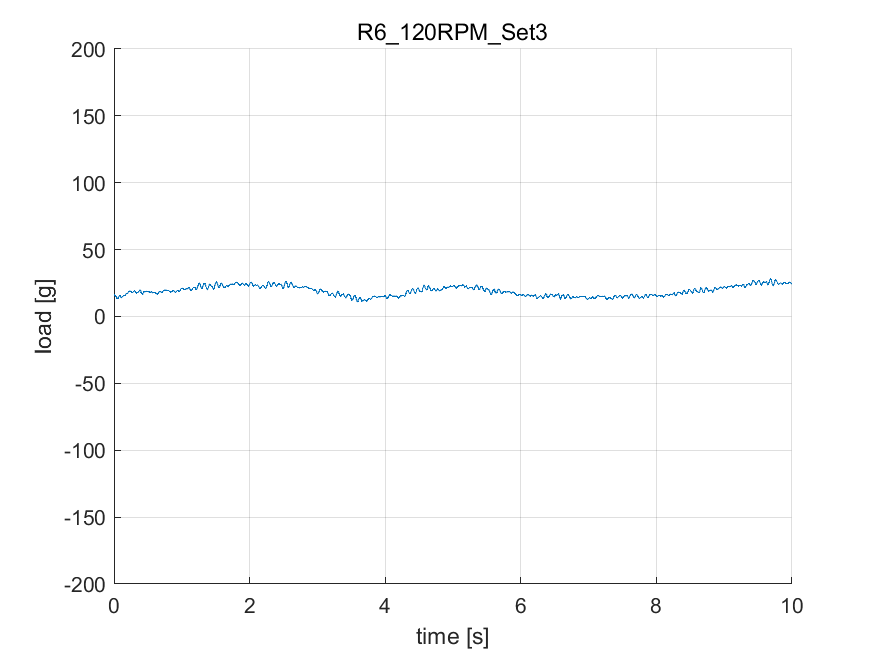

Supplement: Supplementary file 1 — Supplementary Information. [file 41598_2022_25181_MOESM1_ESM.zip › Loadcell_data_graph/R6_120RPM_Set3.png]

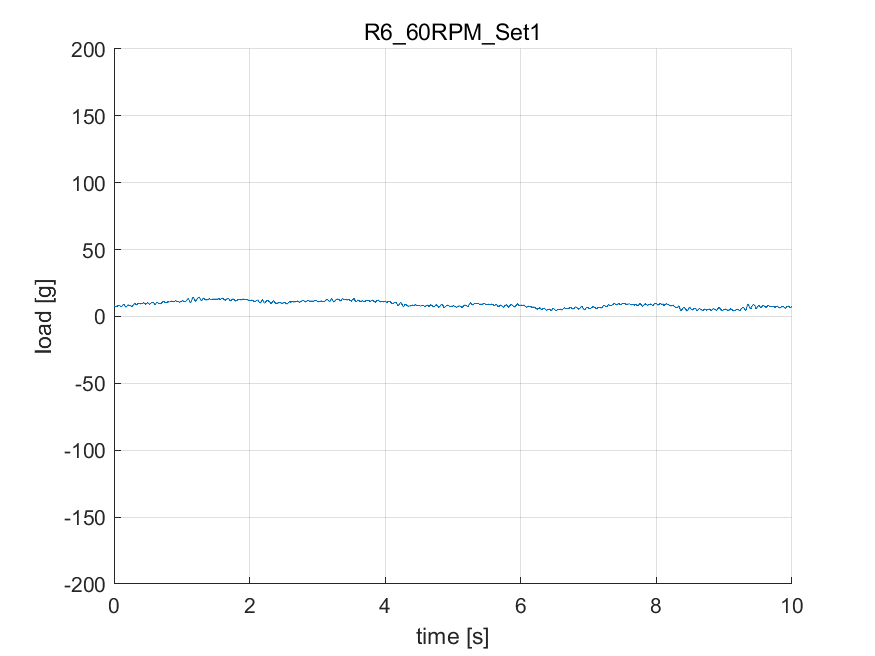

Supplement: Supplementary file 1 — Supplementary Information. [file 41598_2022_25181_MOESM1_ESM.zip › Loadcell_data_graph/R6_60RPM_Set1.png]

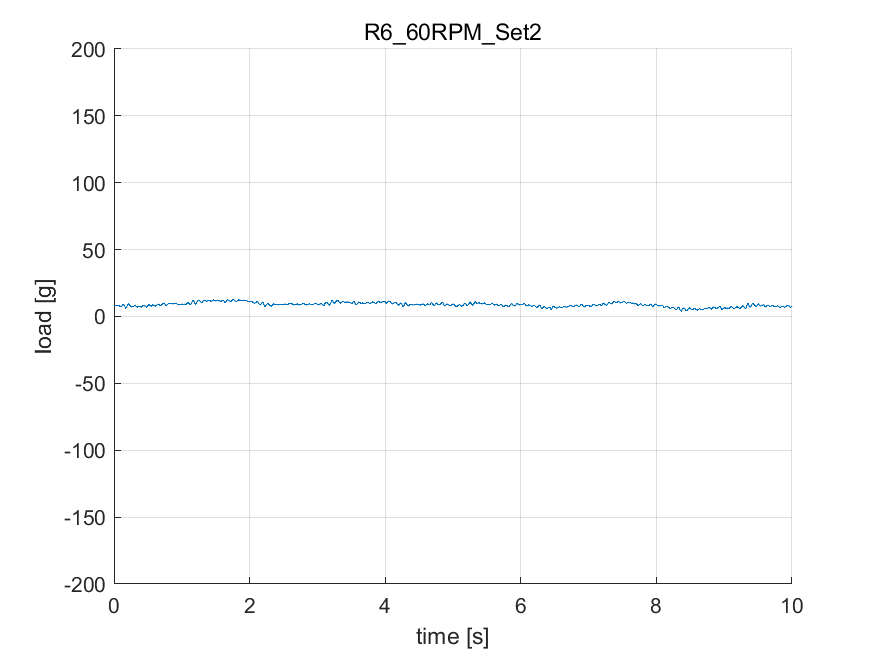

Supplement: Supplementary file 1 — Supplementary Information. [file 41598_2022_25181_MOESM1_ESM.zip › Loadcell_data_graph/R6_60RPM_Set2.png]

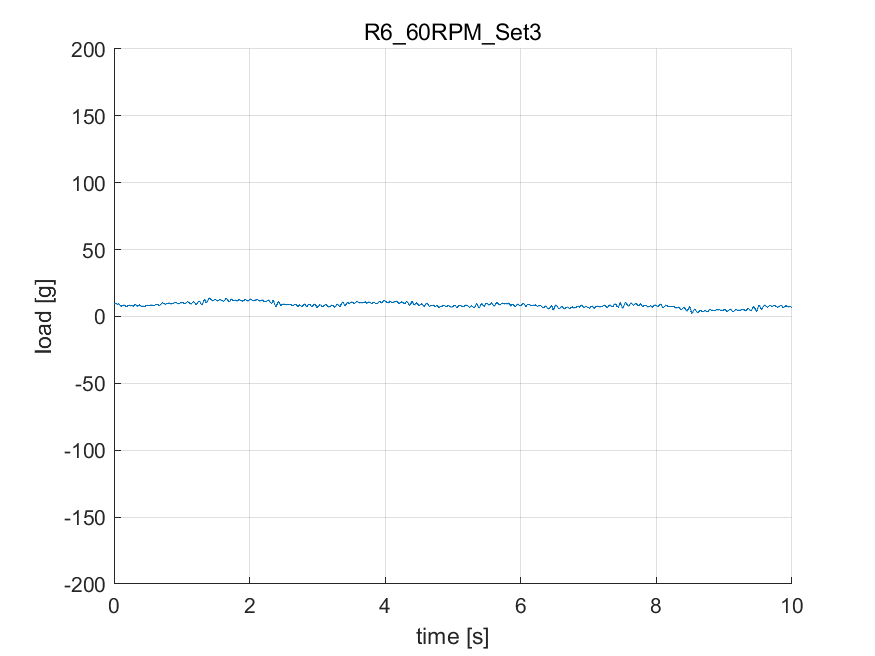

Supplement: Supplementary file 1 — Supplementary Information. [file 41598_2022_25181_MOESM1_ESM.zip › Loadcell_data_graph/R6_60RPM_Set3.png]

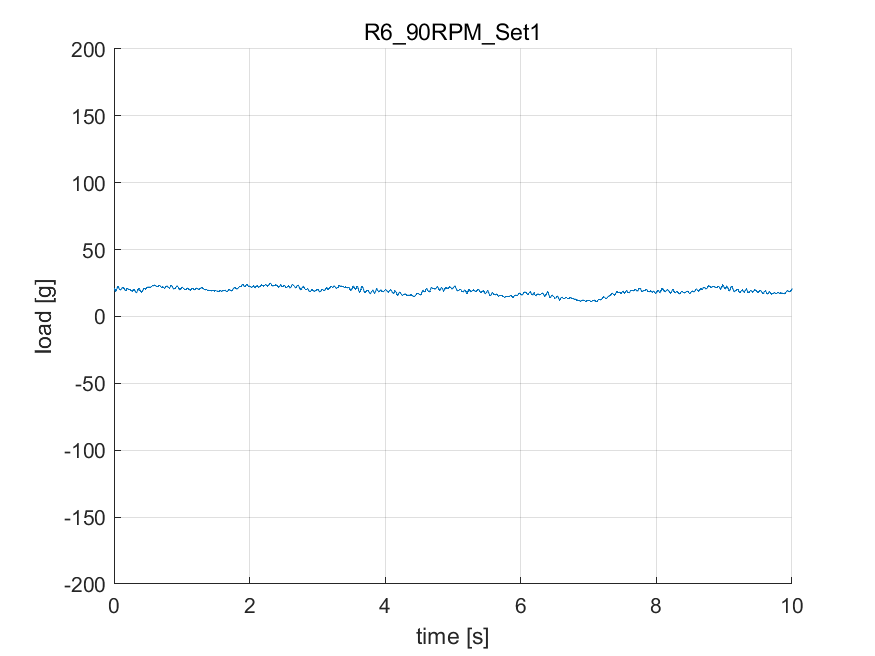

Supplement: Supplementary file 1 — Supplementary Information. [file 41598_2022_25181_MOESM1_ESM.zip › Loadcell_data_graph/R6_90RPM_Set1.png]
